# Supplementary material for: Chlorophyll biosynthesis under the control of arginine metabolism
Source: Cell Rep. 2023 Oct 20;42(11):113265. doi: 10.1016/j.celrep.2023.113265 (PMC10783636; doi:10.1016/j.celrep.2023.113265)
Supplement: Document S1. Figures S1–S4 and Tables S1–S5 [file mmc1.pdf]

Cell Reports, Volume *42*

Supplemental information

Chlorophyll biosynthesis

under the control of arginine metabolism

Éva Kiss, Jana Talbot, Nathan B.P. Adams, Stanislav Opekar, Martin Moos, Jan Pilný, Tatjana Kvasov, Emilia Schneider, Peter Koník, Petr Šimek, and Roman Sobotka

Supplemental information for:

Chlorophyll biosynthesis under the control of arginine metabolism

Éva Kiss[1], Jana Talbot[1#], Nathan B.P. Adams[2,3], Stanislav Opekar[4], Martin Moos[4], Jan Pilný[1], Tatjana Kvasov[2], Emilia Schneider[2], Peter Koník[1,5], Petr Šimek[4], Roman Sobotka[1,5*]

[1]Laboratory of Photosynthesis, Centre Algatech, Institute of Microbiology, Czech Academy of Sciences, 37901 Třeboň, Czech Republic; [2]NanoTemper Technologies, Floessegasse 4, Munich, 81369, Germany; [3]Department of Molecular Biology and Biotechnology, University of Sheffield, Sheffield S10 2TN, United Kingdom; [4]Biology Centre of the Czech Academy of Sciences, Branišovská 1160/31, 370 05 České Budějovice, Czech Republic; [5]Faculty of Science, University of South Bohemia, 37005 České Budějovice, Czech Republic

[#]Present address: Wicking Dementia Research and Education Centre, University of Tasmania, Tasmania, Australia

Supplemental tables

Table S1. The list of *Synechocystis* sp. PCC 6803 strains used in the study

Gene IDs are derived from the cyanobacterial genome database (cyanobase; http://genome.microbedb.jp/cyanobase).

**Strain**	**description**	**reference**
wild type (WT)	*Synechocystis* sp. PCC 6803 GT-P	[S1]
Δ*argD*	Ery^R replacement of *argD* (*slr1022*)	this study
f.argD$^+$/Δ*argD*	3xFLAG-tagged ArgD and Km^R replacement of *psbAII* in Δ*argD*	this study
f.astC$^+$/Δ*argD*	3xFLAG-tagged AstC and Km^R replacement of *psbAII* in Δ*argD*	this study
Δ*cphB*	Sp^R replacement of *cphB* (*slr2001*)	this study
f.argD$^+$/Δ*cphB*	3xFLAG-tagged ArgD and Km^R replacement of *psbAII* in Δ*cphB*	this study
f.gun4$^+$/Δ*gun4*	3xFLAG-tagged Gun4 (Sll0558) and Km^R replacement of *psbAII* in Δ*gun4* (Zeo^R)	[S2]

Table S2. The list of the specific binding partners of f.ArgD

The f.ArgD pull-down was separated by two dimensional blue native/SDS-PAGE (Figure 1B). The most intense protein spots were cut, trypsin-digested and analysed by protein mass-spectrometry.

protein name	gene	locus	protein MW (kDa)	coverage (%)
N-acetylornithine aminotransferase	*argD*	slr1022	46.567	57.6
Cyanophycinase	*cphB*	slr2001	29.389	72.3
Genome Uncoupled 4	*gun4*	sll0558	26.465	74.2
Slr0983	*rfbF*	slr0983	28.793	64.1

Table S2 supplements Figure 1B.

Table S3. **The list of the specific binding partners of f.Gun4**

The f.Gun4 pull-down was separated by SDS-PAGE. After staining the gel (Figure 2C), the most intense protein bands were cut, trypsin-digested and analysed by protein mass-spectrometry.

protein name	gene	locus	protein MW (kDa)	coverage (%)
pyruvate kinase 1	*pyk-1*	sll0587	51.96	44
N-acetylornithine aminotransferase	*argD*	slr1022	46.567	49.7
Genome Uncoupled 4	*gun4*	sll0558	26.465	60
Cyanophycinase	*cphB*	slr2001	29.389	72.3

Table S3 supplements Figure 2A.

Table S4. Metabolic changes caused by ornithine (Orn) or N-acetylornithine (AcOrn)-feeding

Synechocystis grown in BG-11 media were supplemented with 100 µM Orn or AcOrn for 40 min. The intracellular, mM concentrations of the metabolites were determined by LC-MS combined with GC-MS. Control-1, 2 and 3 correspond to the biologically independent samples measured before the addition of Orn or AcOrn. GABA, 2-aminobutyrate; 2-OG, 2-oxoglutarate; Ile, isoleucine; Asn, asparagine; Met, methionine; Glu, glutamate; Phe, phenylalanine; Gln, glutamine; Trp, tryptophan.

	control-1	control-2	control-3	Orn-1	Orn-2	Orn-3	AcOrn-1	AcOrn-2	AcOrn-3
fumarate	0.75	0.5	0.77	0.69	0.41	0.4	0.51	0.37	0.53
succinate	3.15	2.76	3.45	2.37	1.53	1.66	2.47	2.26	2.46
alanine	1.49	1.36	1.8	2.18	2.08	2.05	1.43	1.29	1.52
glycine	0.33	0.25	0.37	0.49	0.39	0.51	0.25	0.28	0.31
GABA	0.04	0.04	0.1	0.02	0.01	0.05	0.03	0.03	0.07
valine	0.24	0.35	0.72	0.2	0.15	0.21	0.11	0.1	0.19
2-OG	0.98	0.72	0.92	0.41	0.3	0.3	0.72	0.61	0.76
Ile	0.22	0.25	0.46	0.18	0.13	0.17	0.12	0.11	0.19
leucine	0.09	0.10	0.20	0.09	0.06	0.08	0.06	0.06	0.09
threonine	0.33	0.31	0.41	0.39	0.38	0.37	0.27	0.29	0.36
serine	1.34	1.43	1.48	1.23	1.19	1.20	1.25	1.27	1.31
proline	0.15	0.25	0.24	8.81	13.76	14.43	0.96	1.53	1.06
Asn	0.17	0.17	0.21	0.25	0.20	0.19	0.19	0.19	0.21
malate	2.98	2.41	3.46	3.05	2.16	2.08	2.50	2.01	2.57
citric	1.66	1.78	1.92	1.45	1.12	1.26	1.45	1.28	1.16
aspartate	0.44	0.58	0.53	0.97	0.82	0.69	0.40	0.41	0.34
Met	0.08	0.09	0.11	0.09	0.08	0.08	0.07	0.08	0.09
Glu	16.61	16.59	18.22	35.27	31.07	29.48	18.06	18.12	20.03
Phe	0.13	0.11	0.14	0.10	0.07	0.07	0.10	0.10	0.12
Gln	0.29	0.44	0.36	0.58	0.56	0.39	0.43	0.39	0.43
Orn	0.06	0.05	0.05	19.63	17.02	16.08	0.32	0.22	0.21
lysine	0.11	0.08	0.15	0.27	0.14	0.17	0.10	0.08	0.10
tyrosine	0.19	0.15	0.21	0.19	0.17	0.14	0.18	0.17	0.18
Trp	0.03	0.03	0.04	0.03	0.02	0.02	0.03	0.04	0.04
arginine	0.07	0.08	0.08	0.71	0.67	0.69	0.09	0.10	0.10
citrulline	0.16	0.13	0.10	5.88	5.63	5.07	0.49	0.52	0.45
AcOrn	n.d.	n.d.	n.d.	0.19	0.15	0.15	n.d.	n.d.	n.d.

Table S4 contains the complete data used for generating the heat map shown in Figure S3A.

Table S5. Oligonucleotides used in the study.

primer	sequence	reference
Primer for the replacement of *argD* with erythromycin resistance cassettes (forward-1):	GGCTTCGGTGAAACGATTTAC	This study
Primer for the replacement of *argD* with erythromycin resistance cassettes (forward-2):	GCATCCCTTAACTTGTTTTTCGGCG ATCGCCACTTTGGTTTG	This study
Primer for the replacement of *argD* with erythromycin resistance cassettes (reverse-1):	CTGCAATCTGATGCGATTATTGAAC AACAGGGGAATAGGTCACG	This study
Primer for the replacement of *argD* with erythromycin resistance cassettes (reverse-2):	GCCGAGCCGGAATGAGCAGA	This study
Primer for the replacement of *cphB* with spectinomycin resistance cassettes (forward-1):	CAACCCCAGCGTCCCGTAAC	This study
Primer for the replacement of *cphB* with spectinomycin resistance cassettes (forward-2):	GAAGTAATCGCAACATCCGCGGAT ATGGCGGTGGGGCTG	This study
Primer for the replacement of *cphB* with spectinomycin resistance cassettes (reverse-1):	GAAGTCGAGGCATTTCTGTCCGGT TGGGAGGATAGGGGCAT	This study
Primer for the replacement of *cphB* with spectinomycin resistance cassettes (reverse-2):	GCGCAACACGCCGACTATC	This study
Primer for cloning *f.argD*$^{+}$/Δ*argD (forward):*	AGCTACATATGACCTATTCCCCTGT TGTT	This study
Primer for cloning *f.argD*$^{+}$/Δ*argD (reverse):*	GGATCAGATCTCAAACCAAAGTGG CGAT	This study
Primer for cloning *f.astC*$^{+}$/Δ*argD (forward):*	CTAGACGCGGCCGCAGCAATTGAA CAAACAGCAATTACA	This study
Primer for cloning *f.astC*$^{+}$/Δ*argD (reverse):*	GAGGACAGATCTTTACGCCCCAAC CACCTTCG	This study
Primer for amplifying FLAG-constructs (forward):	TGTCATCTATAAGCTTCGTG	This study
Primer for amplifying FLAG-constructs (reverse):	ATCCGCCGGCAGACGTTCTTCC	This study
Primer for sequencing FLAG-constructs (forward):	AACTCTCATTAATCCTTTAG	This study
Primer for sequencing FLAG-constructs (reverse):	AGACCGATACCAGGATCTTG	This study

Supplemental figures

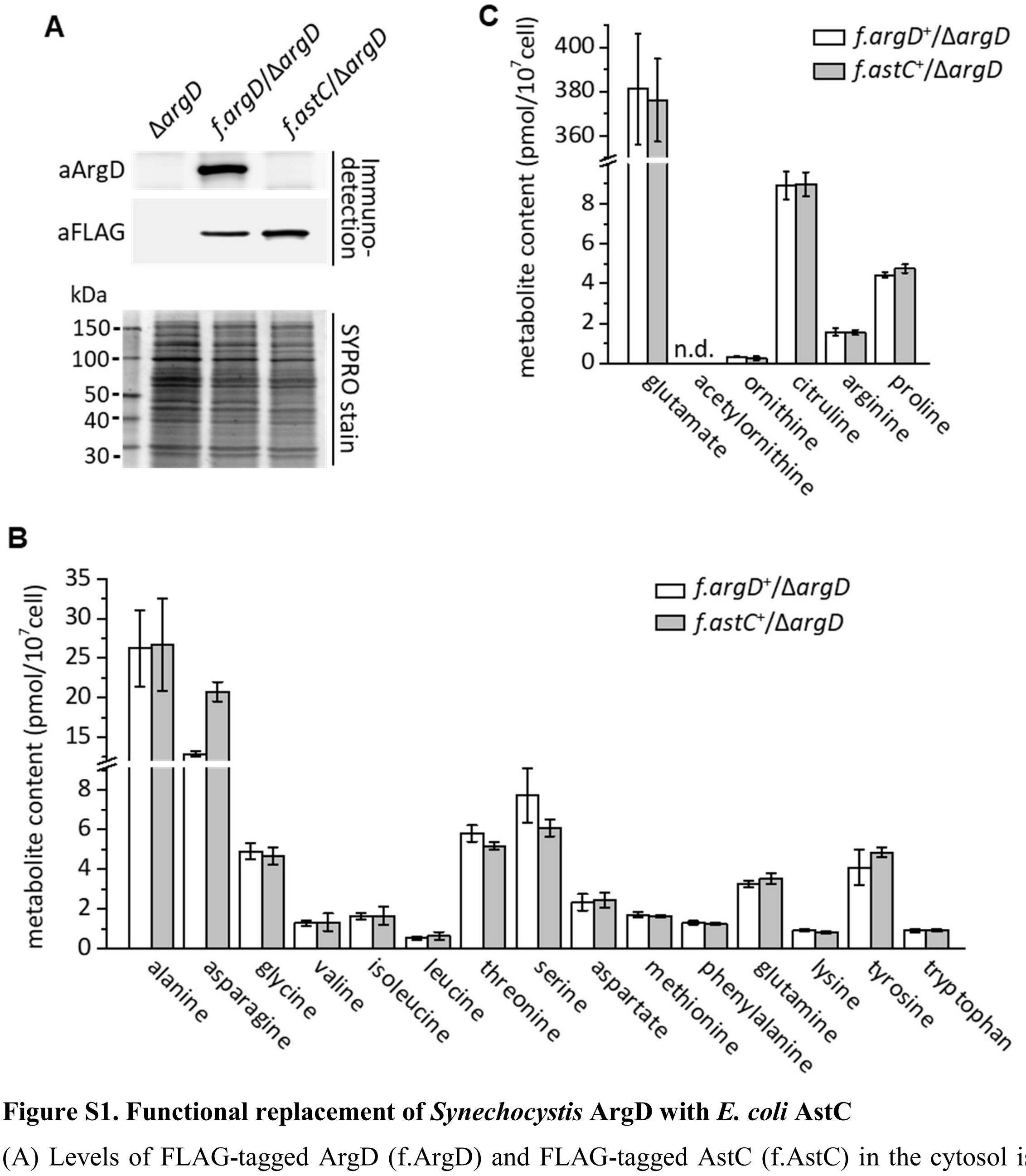

Figure S1. Functional replacement of *Synechocystis* ArgD with *E. coli* AstC

(A) Levels of FLAG-tagged ArgD (f.ArgD) and FLAG-tagged AstC (f.AstC) in the cytosol isolated form the *f.argD*$^+$/Δ*argD* and *f.astC*$^+$/Δ*argD* strains. The f.ArgD and f.AstC proteins were detected using antibody against their 3xFLAG-tag sequence. The SYPRO Orange dye of the SDS gel is shown for loading control.

(B) The cellular content of selected metabolites in *f.argD*$^+$/Δ*argD* (white) and *f.astC*$^+$/Δ*argD* (grey) was determined by LC-MS and GC-MS analysis. The columns and error bars represent the averaged data and standard deviation derived from samples collected from three independent, exponentially grown cultures, respectively.

(C) Cellular content of the Arg metabolism-related amino acids in *f.argD*$^{+}$/Δ*argD* (white) and *f.astC*$^{+}$/Δ*argD* (grey). For details see panel (B). n.d., not detected.

Figure S1 supports Figures 1A, 2C and D by indicating that the f.AstC is expressed in Δ*argD* and it fulfills the enzymatic function of ArgD in the biosynthesis of Orn without significantly modifying the accumulation of amino acids.

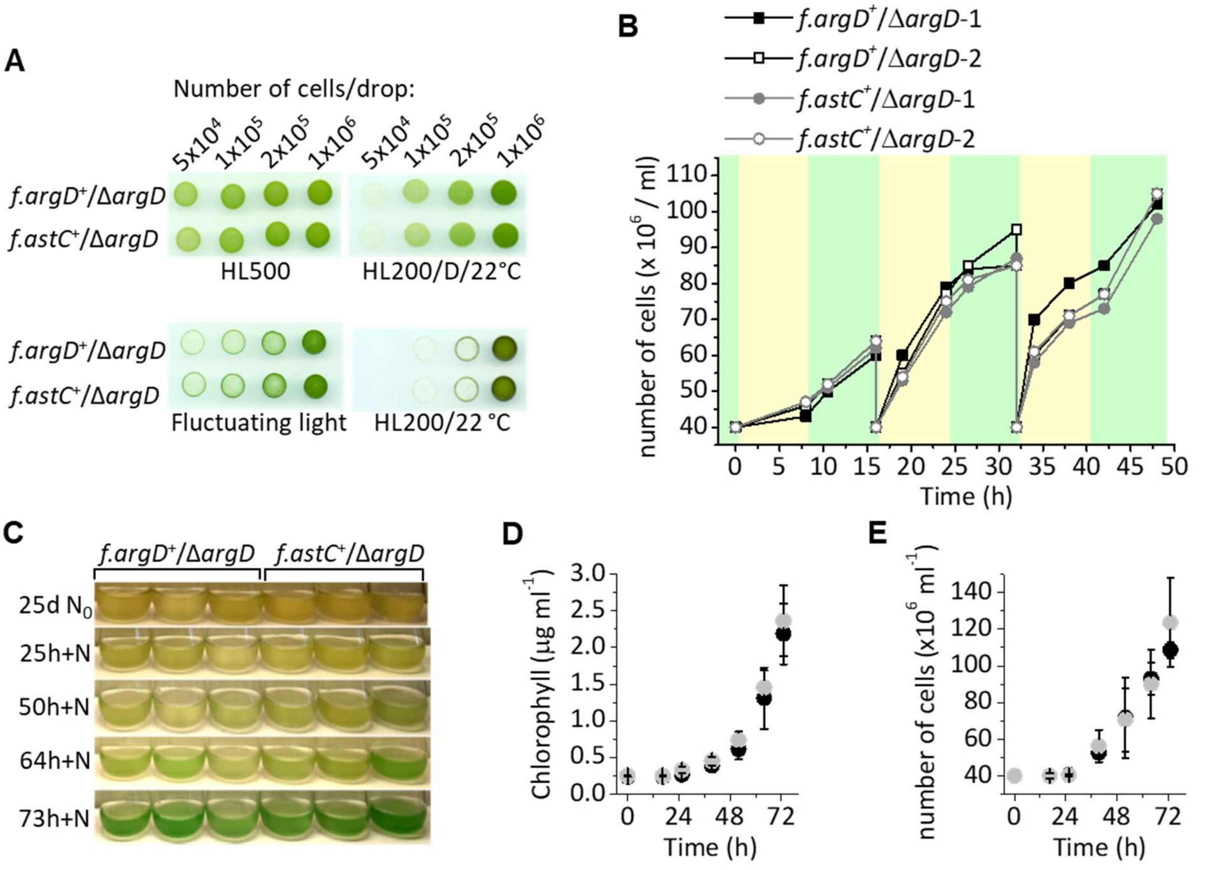

Figure S2. Comparison of the *f.argD*$^+$/Δ*argD* and *f.astC*$^+$/Δ*argD* strains under various physiological conditions

(A) Growth of *f.argD*$^+$/Δ*argD* and *f.astC*$^+$/Δ*argD* monitored in plate-drop experiments using the following conditions: HL500, continuous illumination with 500 µmol m^{-2} s^{-1}; fluctuating light, 5 min dark / 5 min 500 µmol m^{-2} s^{-1}; HL200/D/22 °C and HL200/22 °C, 200 µmol m^{-2} s^{-1} light intensity combined with 22 °C cold stress during diurnal growth (12/12 h) or continuous light, respectively.

(B) Proliferation of *f.argD*$^+$/Δ*argD* and *f.astC*$^+$/Δ*argD* cells in liquid medium during repeated changes of N-rich (green background) and N-less (yellow background) media. When the medium was changed to the N-less, the amount of cells in every culture was adjusted to 40x10^6 cells / ml. The duplicate cultures are designated by 1 and 2.

(C) Resuscitation of *f.argD*$^+$/Δ*argD* and *f.astC*$^+$/Δ*argD* cultures. Dormancy was induced by 25 d of N-deprivation. After 25 d in N_0 media the cells were washed to BG-11 (+N) to reach 40 x 10^6 cells/ml concentration. Cultures were monitored at the indicated time points.

(D) and (E) are the averaged Chl and cell contents of the *f.argD*$^+$/Δ*argD* (black symbols) and *f.astC*$^+$/Δ*argD* (grey symbols) cultures described in panel (C), respectively.

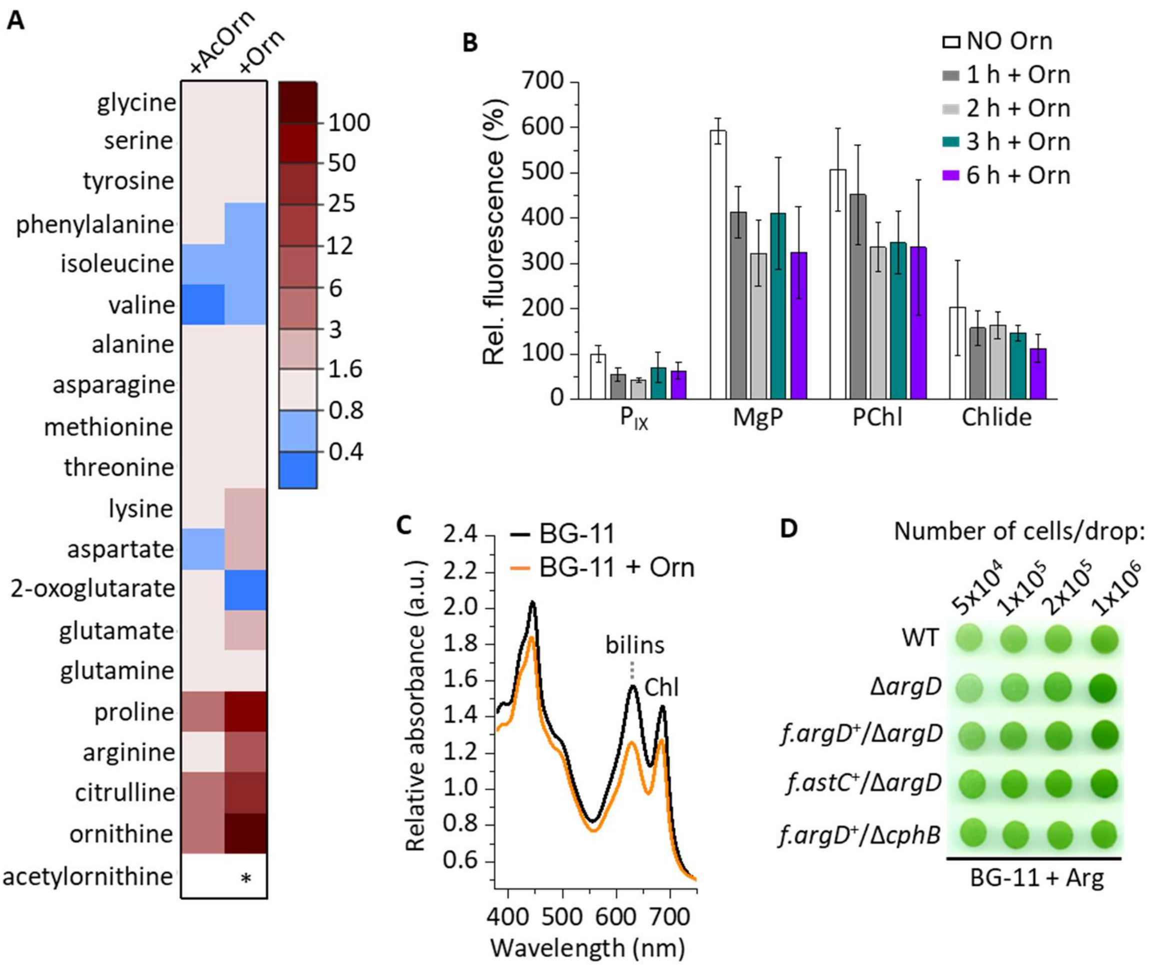

Figure S3. Response to Arg, N-acetylornithine (AcOrn) or ornithine (Orn) feeding

(A) *Synechocystis* cultures were supplemented with 100 µM AcOrn or Orn for 40 min. The cellular content of the selected metabolites was determined by LC-MS and GC-MS analysis, and is shown after normalization to the control sample (without the added ornithines). The heat map was generated from the averaged data obtained from three independent biological experiments. *, AcOrn was not detected in the control sample, even after AcOrn-feeding; only after the addition of Orn (163 ± 26 µM). The complete dataset is shown in the Table S4.

(B) Changes in the relative abundances of the biosynthetic intermediates of Chl in Orn-fed WT cells. Samples were taken before (NO Orn) and after the addition of 1 mM Orn at the indicated time points.

(C) The effect of Orn on the pigmentation of *Synechocystis* WT cell. The cell absorption spectra were measured on same amount of cells cultivated for 3 d in BG-11 with (orange line) or without (black line) 1 mM Orn.

(D) The effect of Arg on the growth of the *Synechocystis* strains used in the study.

Figure S3 complements Figures 3, and explains the choice of Orn feeding for the purpose of perturbation of Arg metabolism and its effect on the *in vivo* accumulation of the Gun4-ArgD-CphB complex.

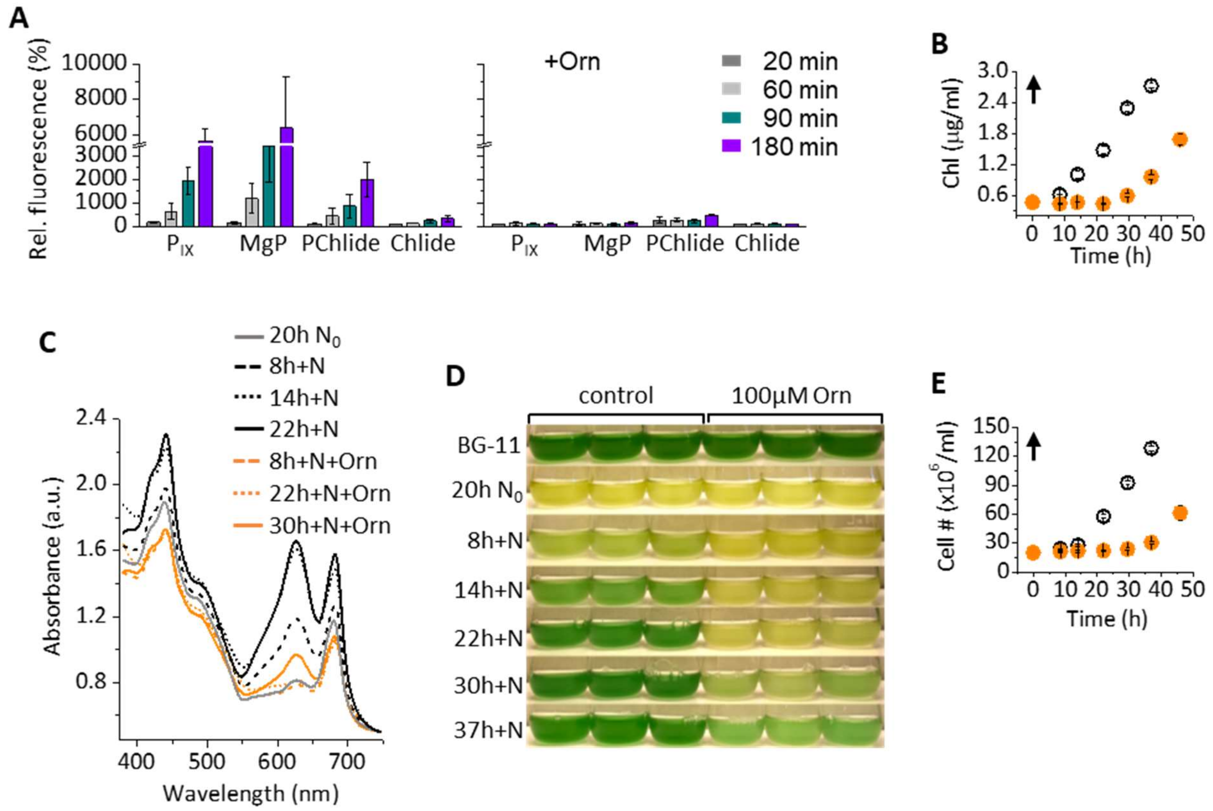

Figure S4. Orn postpones the recovery of *Synechocystis f.argD*$^{+}$/Δ*argD* cells from nitrogen-deprivation

(A) The relative amounts of Chl precursors were measured during N-repletion of 20 h N-starved *f.argD*$^{+}$/Δ*argD* cells. The amounts before the addition of 1mM $NaNO_3$ or 1mM $NaNO_3$ + 100 µM Orn (+Orn) were taken as 100 %. Columns and error bars represent the averaged data and standard deviation of three independent experiments, respectively.

(B) Accumulation of Chl in *f.argD*$^{+}$/Δ*argD* cultures during N-repletion in the absence (black) or presence (orange) of 100 µM Orn. Error bars represent standard deviation of three independent experiments. The time of N-upshift is marked by upward arrows.

(C) Accumulation of phycobilin (625nm) and Chl-binding (682 nm) proteins in the 20 h N-starved *f.argD*$^{+}$/Δ*argD* cells before (20h N_0) and after the addition of 1 mM $NaNO_3$ without (black) or with (orange) 100 µM Orn. The *in vivo* absorption spectra were measured on the same amount of cells at indicated time points.

(D) The cultures before (BG-11) and after (20 h N_0) N-starvation, and during the re-greening without (control) or with 100 µM Orn (as described in panel A) were photographed at indicated times.

(E) Changes in the concentration of cells in the cultures described in panel A and B.

Figure S4 supplements Figure 4, and shows that similarly to the WT control, the *f.argD*$^{+}$/Δ*argD* cultures exhibit delayed re-greening in the presence of Orn.

Supplemental references

S1. Tichý, M., Bečková, M., Kopečná, J., Noda, J., Sobotka, R., and Komenda, J. (2016). Strain of *Synechocystis* PCC 6803 with aberrant assembly of photosystem II contains tandem duplication of a large chromosomal region. Front Plant Sci *7*, 648. 10.3389/fpls.2016.00648.

S2. Sobotka, R., Dühring, U., Komenda, J., Peter, E., Gardian, Z., Tichý, M., Grimm, B., and Wilde, A. (2008). Importance of the cyanobacterial GUN4 protein for chlorophyll metabolism and assembly of photosynthetic complexes. J Biol Chem *283*, 25794-25802. 10.1074/jbc.M803787200.
