# Supplementary material for: Chlorophyll biosynthesis under the control of arginine metabolism
Source: Cell Rep. 2023 Oct 20;42(11):113265. doi: 10.1016/j.celrep.2023.113265 (PMC10783636; doi:10.1016/j.celrep.2023.113265)
Supplement: Document S2. Article plus supplemental information [file mmc2.pdf]

Cell Reports

Report

Chlorophyll biosynthesis under the control of arginine metabolism

Graphical abstract

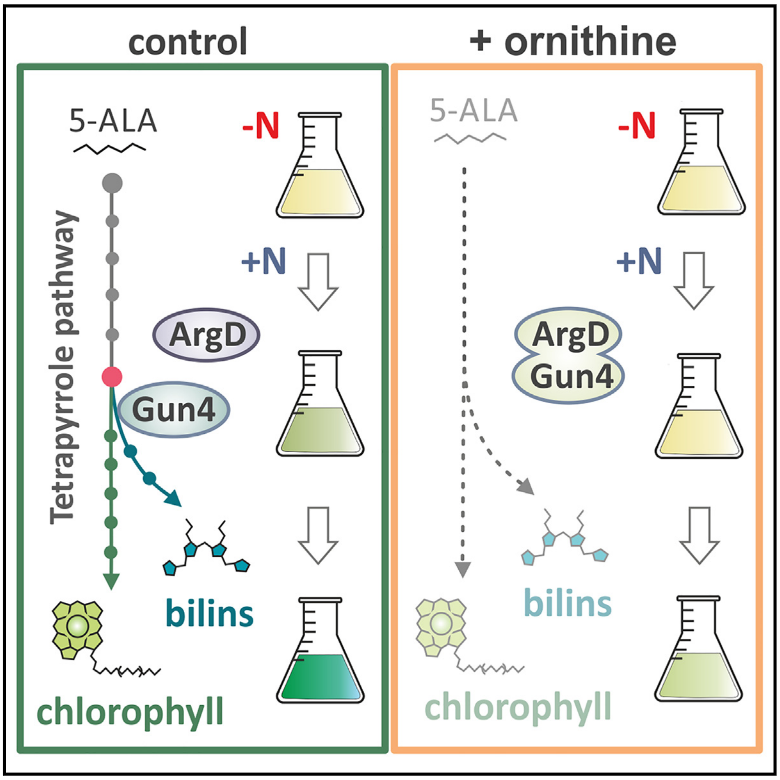

Highlights

- ArgD of arginine pathway interacts with the Gun4 protein of chlorophyll biosynthesis
- Ornithine stimulates the ArgD-Gun4 interaction and inhibits tetrapyrrole synthesis
- The inhibition by ornithine is released in a strain lacking the ArgD-Gun4 complex

Authors

Éva Kiss, Jana Talbot, Nathan B.P. Adams, ..., Peter Koník, Petr Šimek, Roman Sobotka

Correspondence

sobotka@alga.cz

In brief

Kiss et al. demonstrate that the synthesis of the main photosynthetic pigment, chlorophyll, and the metabolism of arginine (managing N-stockpile) are functionally coupled via protein-protein interactions in cyanobacteria.

Kiss et al., 2023, Cell Reports *42*, 113265
November 28, 2023 © 2023 The Author(s).
https://doi.org/10.1016/j.celrep.2023.113265

CellPress

Cell Reports

CellPress
OPEN ACCESS

Report

Chlorophyll biosynthesis under the control of arginine metabolism

Éva Kiss,[1] Jana Talbot,[1,6] Nathan B.P. Adams,[2,3] Stanislav Opekar,[4] Martin Moos,[4] Jan Pilný,[1] Tatjana Kvasov,[2] Emilia Schneider,[2] Peter Koník,[1,5] Petr Šimek,[4] and Roman Sobotka[1,5,7,*]

[1]Laboratory of Photosynthesis, Centre Algatech, Institute of Microbiology, The Czech Academy of Sciences, 37901 Třeboň, Czech Republic
[2]NanoTemper Technologies, Floessegasse 4, 81369 Munich, Germany
[3]Department of Molecular Biology and Biotechnology, University of Sheffield, Sheffield S10 2TN, UK
[4]Biology Centre of the Czech Academy of Sciences, Branišovská 1160/31, 370 05 České Budějovice, Czech Republic
[5]Faculty of Science, University of South Bohemia, 37005 České Budějovice, Czech Republic
[6]Present address: Wicking Dementia Research and Education Center, University of Tasmania, Hobart, TAS, Australia
[7]Lead contact
*Correspondence: sobotka@alga.cz

https://doi.org/10.1016/j.celrep.2023.113265

SUMMARY

In natural environments, photosynthetic organisms adjust their metabolism to cope with the fluctuating availability of combined nitrogen sources, a growth-limiting factor. For acclimation, the dynamic degradation/synthesis of tetrapyrrolic pigments, as well as of the amino acid arginine, is pivotal; however, there has been no evidence that these processes could be functionally coupled. Using co-immunopurification and spectral shift assays, we found that in the cyanobacterium *Synechocystis* sp. PCC 6803, the arginine metabolism-related ArgD and CphB enzymes form protein complexes with Gun4, an essential protein for chlorophyll biosynthesis. Gun4 binds ArgD with high affinity, and the Gun4-ArgD complex accumulates in cells supplemented with ornithine, a key intermediate of the arginine pathway. Elevated ornithine levels restricted *de novo* synthesis of tetrapyrroles, which arrested the recovery from nitrogen deficiency. Our data reveal a direct crosstalk between tetrapyrrole biosynthesis and arginine metabolism that highlights the importance of balancing photosynthetic pigment synthesis with nitrogen homeostasis.

INTRODUCTION

In natural environments, the fluctuating combined nitrogen (N) source is a growth-limiting factor. To cope with this constraint, photosynthetic organisms have to adjust their metabolism to the availability of N. For acclimation, the dynamic degradation/synthesis of tetrapyrrolic pigments (chlorophyll [Chl], bilins, hemes) as well as of the amino acid arginine (Arg) are evidently pivotal. Arg metabolism is in aid of balancing N availability for anabolic processes in a fluctuating environment.[1] The surplus N is primarily used for the synthesis of Arg that can be stockpiled in storage material, such as cyanophycin, which can be metabolized during N deficiency.[2] At the same time, N fluctuation causes extensive changes in the metabolism of tetrapyrroles. At low N, the accumulation of Chl and bilins is predominantly downregulated in a process called chlorosis or bleaching.[3,4] Since these tetrapyrroles are essential co-factors for photosynthetic apparatus, bleached cyanobacterial cells stop their photosynthetic activity.[5] When N becomes available, pigment synthesis is reactivated (regreening) for the biogenesis of photosynthetic machinery to allow photoautotrophic growth.[5]

Oxygenic phototrophs synthesize the main photosynthetic pigment, Chl, together with other tetrapyrroles via a common, branched pathway.[6] The tetrapyrrole pathway must be tightly regulated in virtually any type of organism since protoporphyrins and other pyrrolic intermediates are highly phototoxic.[7] In oxygenic phototrophs, the regulation of the tetrapyrrole pathway appears to be a particularly complicated task, as the pathway is branched and high quantities of end products (Chl, heme, bilins) are required for photosynthesis.[6] A sophisticated control mechanism(s) must evolve to counterbalance the pathway with other processes in the cell. In this way, the amounts of tetrapyrrole end products can vary depending on developmental stage and/or environmental conditions, and the accumulation of the phototoxic biosynthetic intermediates can be tightly coordinated.[6] How this regulation is accomplished remains mostly unclear. However, the main regulatory mechanisms appear linked to the formation of the rate-limiting precursor 5-aminolevulinc acid (5-ALA) and the branching between Chl and heme pathways.[8,9] At this branchpoint, the magnesium chelatase (MgCh) and ferrochelatase enzymes compete for the same protoporphyrin IX (P_{IX}) substrate.

The Gun4 protein has been recognized as a critical factor for the synthesis of Chl in cyanobacteria, as well as in algae and plants, and has been extensively studied by various biochemical and physiological approaches.[10–17] It is functionally linked to MgCh; however, *in vivo* characterization of Gun4 mutants revealed complex changes in tetrapyrrole metabolism far beyond

© 2023 The Author(s).
This is an open access article under the CC BY license (http://creativecommons.org/licenses/by/4.0/).

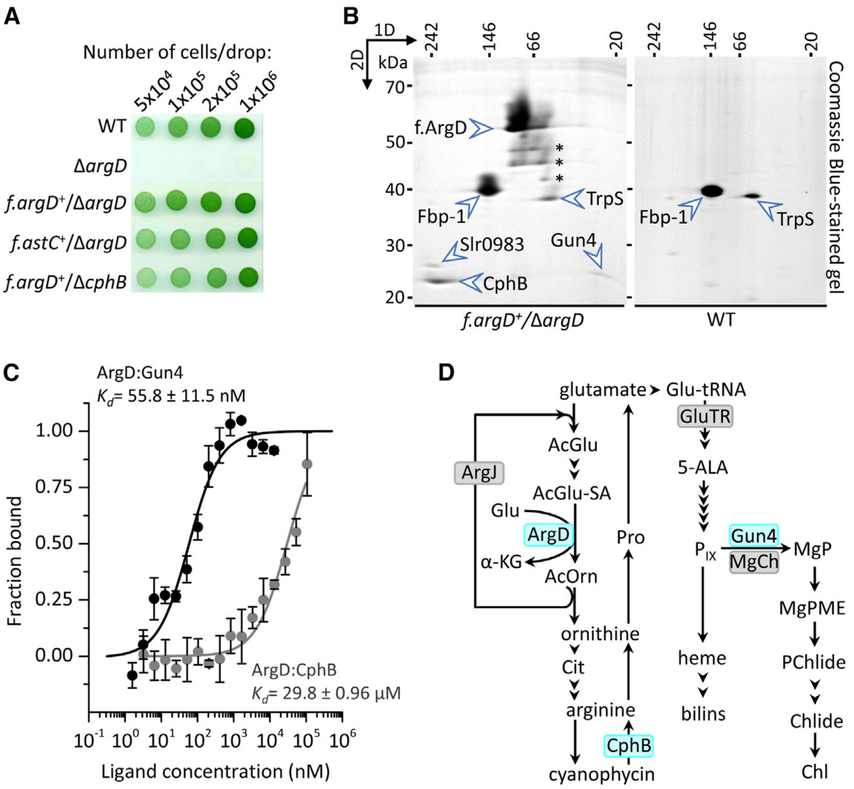

Figure 1. The Gun4 protein interacts with enzymes involved in Arg metabolism

(A) Photoautotrophic growth of *Synechocystis* strains constructed for anti-FLAG pull-downs.

(B) 2D BN/SDS-PAGE separation of *f.argD*$^+$/Δ*argD* and control (WT) pull-downs. The indicated protein dots were identified by MS (Table S2). Protein spots indicated by stars were identified as f.ArgD and most likely represent partially degraded fragments of f.ArgD.

(C) Quantitative analysis of the binding affinities of ArgD to CphB and Gun4. Recombinant proteins were analyzed with a spectral shift binding assay, where 5 nM labeled ArgD was titrated with CphB (gray symbols) or Gun4 (black symbols). Symbols and error bars represent the average data of three independent experiments and their standard deviation; the determined K_d values are indicated.

(D) The main steps of Arg metabolism and the biosynthesis of tetrapyrroles according to Flores et al.[29] and Sobotka[30] and references therein. The interacting protein partners found in this study are designated by blue boxes. AcGlu, N-acetyl-glutamate; AcGlu-SA, AcGlu semialdehyde; α-KG, α-ketoglutarate; AcOrn, N-acetylornithine; Cit, citrulline; Pro, proline; Glu-tRNA, glutamyl-tRNA; GluTR, Glu-tRNA reductase; MgPME, Mg protoporphyrin IX monomethyl ester; PChlide, divinyl protochlorophyllide *a*; Chlide, monovinyl chlorophyllide *a*.

what is expected from the altered MgCh activity.[16–23] These results imply that Gun4 can regulate tetrapyrrole biosynthesis in multiple ways.

Gun4 binds tightly to the ChlH subunit of MgCh, forming a membrane-localized complex, which is expected to be the site of Mg-P_{IX} (MgP) synthesis.[12,24,25] However, a fraction of Gun4 is localized in the cytosol/stroma.[12,24,25] Here, we show that in the cyanobacterium *Synechocystis* sp. PCC 6803 (hereafter *Synechocystis*), the soluble N-acetylornithine aminotransferase (ArgD) and cyanophycinase (CphB) enzymes interact with Gun4. CphB is a peptidase that breaks down the cyanophycin biopolymer into β-Asp-Arg dipeptides.[26] It is categorically found in cyanophycin-containing bacteria and is apparently dispensable in *Synechocystis*.[27] On the other hand, ArgD is an essential enzyme in Arg biosynthesis from bacteria to plants. Our study shows that ArgD binds Gun4 with high affinity and that the accumulation of the Gun4-ArgD complex is modulated by the cellular level of ornithine (Orn), a central intermediate of Arg biosynthesis. High levels of Orn inhibit Chl biosynthesis via an ArgD-dependent mechanism, supporting the role of the Gun4-ArgD complex in a cross-talk between the Arg and tetrapyrrole pathways.

RESULTS

The Gun4 protein interacts with enzymes involved in Arg metabolism

To better understand the metabolism of N in *Synechocystis*, we focused on the Arg metabolic pathway playing a central role in N homeostasis. Specifically, we used anti-FLAG pull-downs to identify protein interactors of enzymes involved in Arg biosynthesis. We constructed a strain expressing 3×FLAG-tagged ArgD (f.ArgD) from the constitutive *psbAII* promoter while lacking the native enzyme (*f.argD*$^+$/Δ*argD* strain; see Table S1). Unlike the Δ*argD* mutant, which is an Orn auxotroph,[28] the *f.argD*$^+$/Δ*argD* mutant proliferated without Orn supplement, showing growth and pigmentation comparable with the wild-type (WT) strain (Figure 1A).

The f.ArgD enzyme was isolated from the soluble fraction according to Koskela et al.,[31] and the obtained eluate was separated on a 2D blue native (BN)/SDS-PAGE together with a WT control. The resulting gel was stained, and the visible protein spots were identified by mass spectrometry (MS) (Figure 1B; Table S2). Tryptophanyl t-RNA synthetase (TrpS) and fructose-1,6-biphosphatase (Fbp-1) were evident contaminants, eluted from the FLAG resin also when using the WT control. On the other hand, despite their detachment from f.ArgD during the separation by BN, Slr0983 (hypothetical glucose-1-phosphate cytidylyltransferase homolog), CphB, and Gun4 were identified as specific co-eluates of f.ArgD (Figure 1B). CphB (29.4 kDa) migrated like a large oligomer (>200 kDa), which was more intensely stained than the spot of the apparently monomeric Gun4 (26.6 kDa). The mass of f.ArgD (49.8 kDa) on the BN gel appeared at about 100 kDa, indicating a dimer consistent with other studies.[32] Given the well-established and critical role of Gun4 in Chl biosynthesis,[24] we focused on the interaction of Gun4 with enzymes involved in Arg metabolism (ArgD and CphB).

To assess the *in vitro* affinities of *Synechocystis* ArgD to CphB and Gun4, we expressed these proteins in *Escherichia coli* (*E. coli*) as 6×His-tagged variants. After purification on a nickel column, recombinant proteins were subjected to isothermal spectral shift assays, in which a standard concentration of labeled ArgD was titrated with CphB or with Gun4. Fitting of the resultant binding isotherms revealed K_d values of 29.8 ± 0.96 μM and 55.8 ± 11.5 nM for the titration of ArgD with CphB and Gun4, respectively (Figure 1C). These results indicate that even though CphB and ArgD both participate in Arg metabolism (see Figure 1D), the *in vitro* binding affinity of ArgD to Gun4 is much stronger than to CphB.

Although Gun4 was recognized in a membrane-localized complex,[24,25] herein, it was shown to interact with the strictly soluble ArgD protein.[33] To confirm the presence of Gun4 in a cytosolic enzyme complex, the co-purification assay was repeated using a cytosolic extract of a *Synechocystis* strain expressing 3× FLAG-tagged Gun4 (f.Gun4). *In vivo* activity of Gun4 has been shown previously to be unaffected by the addition of FLAG tag.[25] The obtained f.Gun4 and control WT eluates were separated by SDS-PAGE and stained, and the most intense protein bands were analyzed by proteomic MS. Pyruvate kinase 1 (Pyk-1), ArgD, and CphB were the most abundant, specific co-eluates of f.Gun4 (Figure 2A; Table S3). The interaction of Gun4 with CphB was analyzed by *in vitro* spectral shift assays, revealing a K_d of 2.16 ± 0.64 μM (Figure 2B). This value indicates a ∼40 times weaker binding affinity of Gun4 to CphB compared with ArgD (Figure 1C).

ArgD strongly binds Gun4 *in vitro* (Figure 1C). Nevertheless, the relatively high level of CphB in the f.ArgD and f.Gun4 pull-downs (Figures 1B, 2A, 2C, and 2D) raises the question of whether CphB is important for the Gun4-ArgD interaction *in vivo*. To address this, we purified f.ArgD from the *cphB*-deletion background (Δ*cphB*) that shows no phenotypic changes during photoautotrophic growth[27] (see also Figure 1A). Since co-purification of Gun4 with f.ArgD was apparently not affected by the absence of CphB (Figures 2C and 2D), we concluded that CphB is not required for the accumulation of the Gun4-ArgD complex *in vivo*.

While Gun4 is specific for oxygenic phototrophs, ArgD is a housekeeping enzyme in virtually any organism. To investigate the role of the Gun4-ArgD complex in *Synechocystis*, we replaced the ArgD enzyme with its FLAG-tagged homolog (AstC) from *E. coli.* The resulting strain (*f.astC*$^+$/Δ*argD*) expressed the f.AstC enzyme to a level comparable to f.ArgD (Figure S1A) while showing no alteration in the accumulation of amino acids including Orn and Arg (Figures S1B and S1C). The purified f.AstC, however, did not co-elute with either Gun4 or CphB (Figures 2C and 2D), implying that the *E. coli* enzyme does not bind these proteins *in vivo*. The *f.astC*$^+$/Δ*argD* strain showed photoautotrophic growth similar to *f.argD*$^+$/Δ*argD* (Figure 1A) and exhibited no apparent phenotype under the various physiological conditions tested (Figures S2A–S2E).

Orn triggers the formation of the Gun4-ArgD complex and reduces the steady states of Chl precursors

Although the interaction of Gun4 with enzymes participating in Arg metabolism was not detectable in *f.astC*$^+$/Δ*argD*, no apparent growth defect was caused by the absence of these protein assemblages (Figures 1A and S2A–S2E). However, it is possible that Gun4-ArgD is important for regulation during severe or prolonged shifts in Arg metabolism, and such conditions are difficult to mimic in laboratory. As an alternative, we monitored the phenotype of studied strains after feeding with biosynthetic intermediates of Arg. Using metabolic profiling, we checked first the ability of *Synechocystis* to uptake N-acetylornithine (AcOrn), the enzymatic product of ArgD. The treatment of WT with 100 μM AcOrn for 40 min did not, however, increase the concentration of this compound in cells above the detection limit, suggesting an inefficient uptake (Figure S3A; Table S4). On the other hand, the same treatment with 100 μM Orn, which is the center intermediate metabolite for both Arg biosynthesis and degradation (see Figure 1D), elevated the concentration of intracellular Orn by three magnitudes. Orn also caused severe changes in the accumulation of other metabolites, including an increase of AcOrn (Figure S3A; Table S4). In line with previous studies,[29,34] Orn effectively redirected metabolic fluxes in the Arg pathway and likely blocked the initial steps of Arg synthesis.[35,36]

Given the well-established role of Gun4 in Chl biosynthesis,[24] we then compared the levels of biosynthetic intermediates of Chl in cells grown with $NaNO_3$ supplement with or without 1 mM Orn. We employed as a negative control the *f.astC*$^+$/Δ*argD* strain, in which the ArgD-Gun4 complex was undetectable (Figures 2C and 2D). While Orn apparently did not affect the pool of Chl precursors in *f.astC*$^+$/Δ*argD*, the *f.argD*$^+$/Δ*argD* and WT strains accumulated significantly ($p < 0.05$) lower relative amounts of the monitored tetrapyrroles (Figures 3A and S3B). Further cultivation in the Orn-containing BG-11 media resulted in decreased amount of bilins and Chl (Figure S3C). Consequently, all the strains used in the study had impaired photoautotrophy in the presence of Orn except for those that did not contain the ArgD enzyme (Δ*argD*, f.*astC*/Δ*argD*; Figure 3B). Orn was converted also to Arg (Figure S3A); however, the direct addition of Arg had a milder or no effect on the photoautotrophic growth (Figure S3D).

To test whether the lower accumulation of Chl precursors in the Orn-fed *f.argD*$^+$/Δ*argD* cells can be related to Gun4-ArgD, we compared the *in vivo* abundance of the complex in cells that were fed with nitrate supplement only or also with Orn before FLAG-affinity purification. Analysis of the obtained f.ArgD pull-downs revealed six times more Gun4 co-purified with f.ArgD from cells pre-treated with 1 mM Orn for 24 h (Figure 3C). It is notable that CphB was also more enriched in the eluate after Orn feeding. In our standard purification protocol, FLAG-tagged proteins are released from the resin under native conditions using FLAG-tag peptide.[31] To exclude the possibility that a fraction of the specifically bound proteins remained attached to the resin after elution, we performed another preparation in which proteins were released from the FLAG resin using SDS instead of FLAG peptide. Using this approach and subsequent immunodetection, we detected an even stronger Orn-stimulated enrichment of Gun4 (>12×) in the pull-down (Figure 3D). In this experiment, we monitored the effect of short-term (1 h) feeding with 100 μM Orn that also substantially increased the amount of f.ArgD-co-purified Gun4 (∼6 times; Figure 3D).

To see the extent of Gun4 sequestered from the membranes to the soluble ArgD-Gun4 complex, we performed western blot

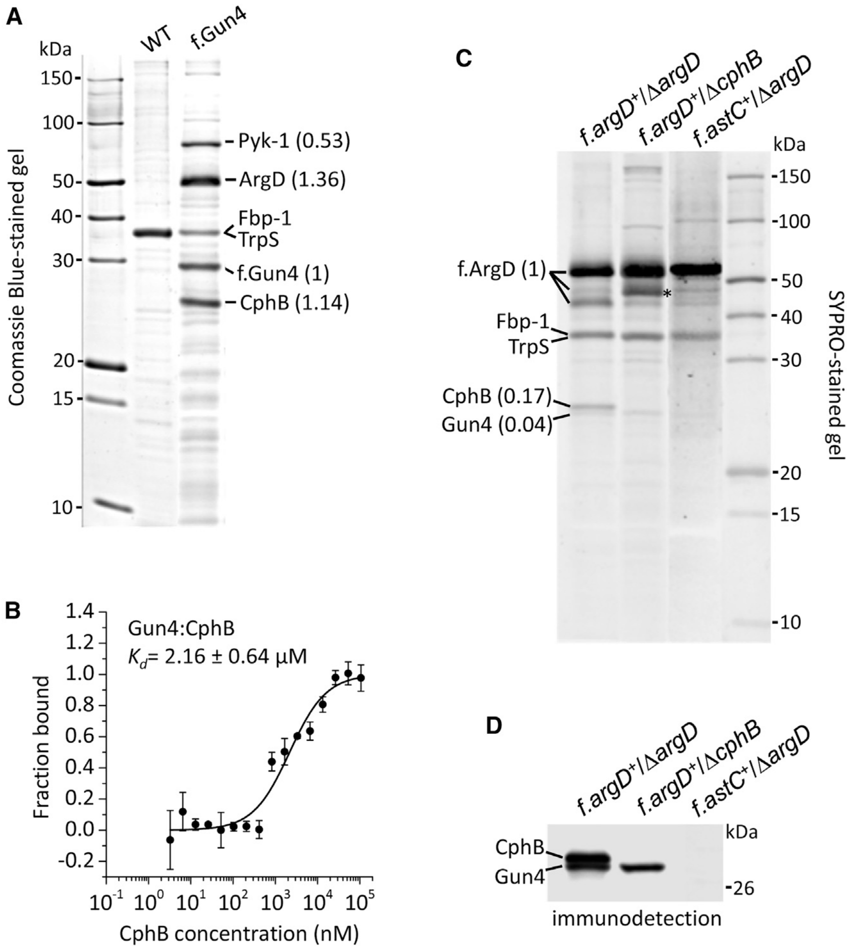

Figure 2. Gun4 is co-isolated with ArgD and CphB but not with the heterologously expressed *E. coli* AstC

(A) SDS-PAGE separation of the f.Gun4 and control (WT) pull-downs. The indicated proteins were identified by MS (Table S3). The molar ratios of co-eluted proteins, calculated from raw band intensities normalized to the molecular weights, are shown in brackets.

(B) Recombinant Gun4 and CphB proteins were subjected to a spectral shift assay, in which 20 nM labeled Gun4 was titrated with CphB. Symbols and error bars represent the average data of three independent experiments and their standard deviation; the obtained K_d value is shown.

(C) Protein pull-downs prepared from indicated strains were separated on SDS-PAGE and stained. Asterisk indicates the native ArgD that was present in the Δ*cphB* background. The molar ratios of co-eluted proteins are also indicated (see A).

(D) The SDS-PAGE gel shown in (C) was blotted to a polyvinylidene fluoride (PVDF) membrane and probed with specific antibodies against Gun4 and CphB.

analysis of the soluble (cytoplasm [C]) and insoluble (membrane [M]) fractions of cells with or without Orn treatment. In fact, a higher portion of Gun4 could be detected from the soluble fraction of the Orn-treated compared with the untreated *f.argD*$^+$/Δ*argD* and WT but not of the *f.astC*$^+$/Δ*argD* cells (Figure 3E). On the other hand, Orn had the opposite effect on the relative distribution of CphB, as it could be detected from the insoluble fraction dependent on Orn treatment.

Orn inhibits *de novo* biosynthesis of tetrapyrroles and postpones recovery from nitrogen deprivation

To clarify whether the lower accumulation of Chl precursors in the presence of additional Orn was due to inhibition of *de novo* tetrapyrrole synthesis, we tested the effect of Orn on regreening cells, in which *de novo* pigment synthesis is cardinal.[37] Bleaching of the cultures was achieved by 20 h of N starvation, and the recovery was induced by the addition of 1 mM $NaNO_3$ alternatively combined with 100 µM Orn. When Orn was omitted from the N supply of the N-starved cultures, Chl precursors were synthetized within 40 min, and after an hour of N upshift, their relative amounts substantially increased in all strains studied (Figures 4A and S4A). However, unlike in the *f.astC*$^+$/Δ*argD* strains, in which Orn did not affect tetrapyrrole synthesis, in the control WT as well as in the *f.argD*$^+$/Δ*argD* strains, the accumulation of Chl precursors (including P_{IX}) was arrested for at least 3 h (Figures 4A and S4A). Consequently, in the Orn-fed WT and *f.argD*$^+$/Δ*argD*, the overall accumulation of Chl was inhibited (Figures 4B and S4B), and in the first 14 h of N upshift, the cell absorption remained comparable to that of the N-starved cultures (Figures 4C and S4C). On the other hand, 14 h of N upshift increased the tetrapyrrole pigment-binding complexes in *f.astC*$^+$/Δ*argD* regardless of the presence of Orn (Figure 4C), while in the Orn-fed WT as well as *f.argD*$^+$/Δ*argD*, the cell absorption peaks at 625 and 682 nm started to increase only after 30 h (Figures 4C and S4C), indicating accumulation of the phycobilin- (625 nm) and Chl-binding (682 nm) photosynthetic proteins that eventually allowed photoautotrophic growth (Figures 4D and S4E). The recovery of the N-starved, bleached WT cultures in the presence of Orn was thus much slower compared with the strain possessing the *E. coli* AstC enzyme (Figure 4E), while it was comparable with the Orn-fed *f.argD*$^+$/Δ*argD* cultures (Figure S4).

DISCUSSION

The Gun4 protein is specific and ubiquitous to oxygenic phototrophs, where it is essential for efficient Chl production. Consistent with the model of active, membrane-bound MgCh,[19] Gun4 was co-isolated with ChlH from thylakoids[24] or total cell extracts[25] but not from the cytosolic fraction (Figure 2A). Prior to this work, the only known protein partner of Gun4 was the ChlH subunit of MgCh. Herein, we identified *Synechocystis* Gun4 in a soluble protein complex(es) consisting of the Arg metabolism-related ArgD and CphB, as well as the Pyk-1

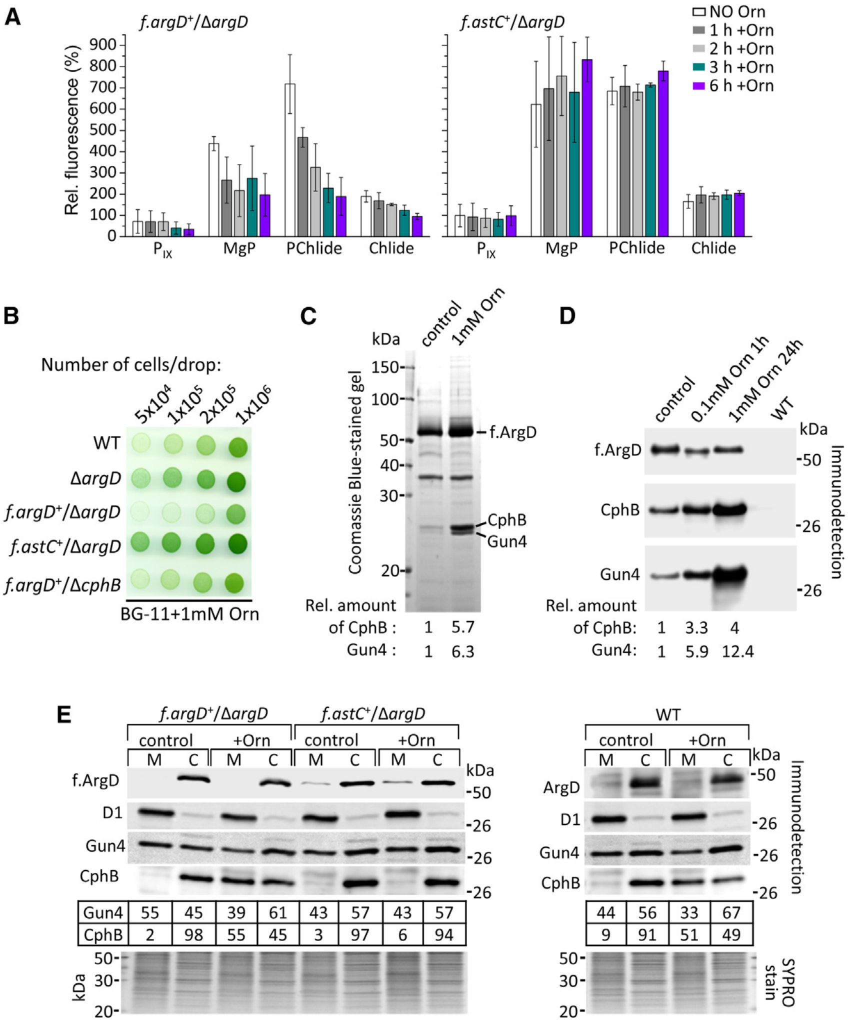

Figure 3. Orn triggers the *in vivo* formation of the Gun4-ArgD complex and reduces the steady states of Chl precursors

(A) The effect of Orn on the steady states of Chl precursors. The columns and error bars represent the averaged data and standard deviation derived from three independent experiments, respectively. The samples were taken before (no Orn) and 1, 2, 3, and 6 h after the addition of 1 mM Orn.

(B) The effect of additional Orn on the photoautotrophic growth of the *Synechocystis* strains used in the study.

(C) Co-immunopurification of Gun4 and CphB with f.ArgD from *Synechocystis* cells that were grown with 18 mM nitrate without (control) or with 24 h treatment with 1 mM Orn. Eluted proteins were separated by SDS-PAGE and stained by Coomassie blue.

(D) Co-immunoprecipitation of the f.ArgD and Gun4 proteins from cells that were grown with 18 mM nitrate without (control) or with Orn supplement. The WT strain was used as control for the specificity of the assay. Proteins were eluted from the anti-FLAG column by 1% SDS and analyzed by western blot.

(C and D) The abundance of CphB and Gun4 relative to ArgD is indicated below each lane (Gun4:ArgD or CphB:ArgD in controls was taken as 1).

(E) Immunoblot analysis of soluble (cytoplasmic [C]) and insoluble (membrane [M]) fractions of the *f.argD*+/Δ*argD*, *f.astC*+/Δ*argD* and WT cells grown in BG-11 without (control) or with 24 h treatment with 1 mM Orn (+Orn). The indicated proteins were detected by antibodies against the whole protein (D1, Gun4, CphB, ArgD) or against the FLAG tag (f.ArgD, f.AstC). Below the immunoblots, the abundance (in percentage) of Gun4 and CphB in the C and M fractions are indicated (total amount in C + M was taken as 100%). The signal of the D1 subunit of photosystem II is shown as a control for the purity of soluble fraction, and the SYPRO Orange stain serves as a loading control.

enzymes (Figure 2A). Apart from Pyk-1, this protein set can be isolated by using both f.ArgD (Figures 1B and 2C) and f.Gun4 (Figure 2A) as bait. The putative interaction of Gun4 with Pyk-1 needs further confirmation and studies. Herein, we confirmed and evaluated the interactions between Gun4-ArgD and ArgD-CphB, as well as Gun4-CphB; however, it is not definite whether we isolate these separate protein-protein interactions or one ternary complex (ArgD-Gun4-CphB). The absence of CphB does not cause obvious phenotypic changes under the photoautotrophic conditions used in our and in previous studies.[27] However, it was previously noted that the Δ*cphB* strain accumulates slightly more bilin-containing light-harvesting complexes.[27] The herein identified interaction of CphB with Gun4 implies that CphB can directly modulate the distribution of Chl and bilin pigments via its interaction with Gun4.

The interaction of ArgD with CphB is likely connected to the regulation of the Arg levels in the cell since both these enzymes contribute to the accumulation of Arg. Additional Orn intensified the interaction of these soluble enzymes; however, at the same time, approximately half of the total amount of CphB could be isolated from the insoluble fraction of the cells. These results are difficult to interpret since the physiological importance of CphB in *Synechocystis* remains enigmatic.

On the other hand, the importance of ArgD as an AcOrn aminotransferase in Arg biosynthesis has been established.[28] Although it was proposed to function also as Orn[38] or gamma aminobutyric acid[39] aminotransferases, these activities are negligible compared to its AcOrn aminotransferase activity.[32] These results imply that ArgD-Gun4 participates in an Arg biosynthesis-dependent regulation of the tetrapyrrole pathway. ArgD bound Gun4 with a K_d of 55.8 ± 11.5 nM (Figure 1C), which is close to the binding constant obtained by a fluorescence quenching measurement for the Gun4-ChlH complex (K_d ~10 nM).[40] These data indicate that the binding affinities of Gun4 to ArgD and ChlH are comparable. We demonstrated that additional Orn, which can also boost the interaction of f.ArgD with Gun4 and CphB (Figures 3C and 3D), halted *de novo* Chl production in the presence, but not in the absence, of *Synechocystis* ArgD (Figure 4A). These results imply an Orn-dependent downregulation of tetrapyrrole biosynthesis that is likely related to the formation of the ArgD-Gun4 complex. ChlH was absent in the herein isolated soluble complexes of Gun4 (Figures 1B, 2A, and 2C). The

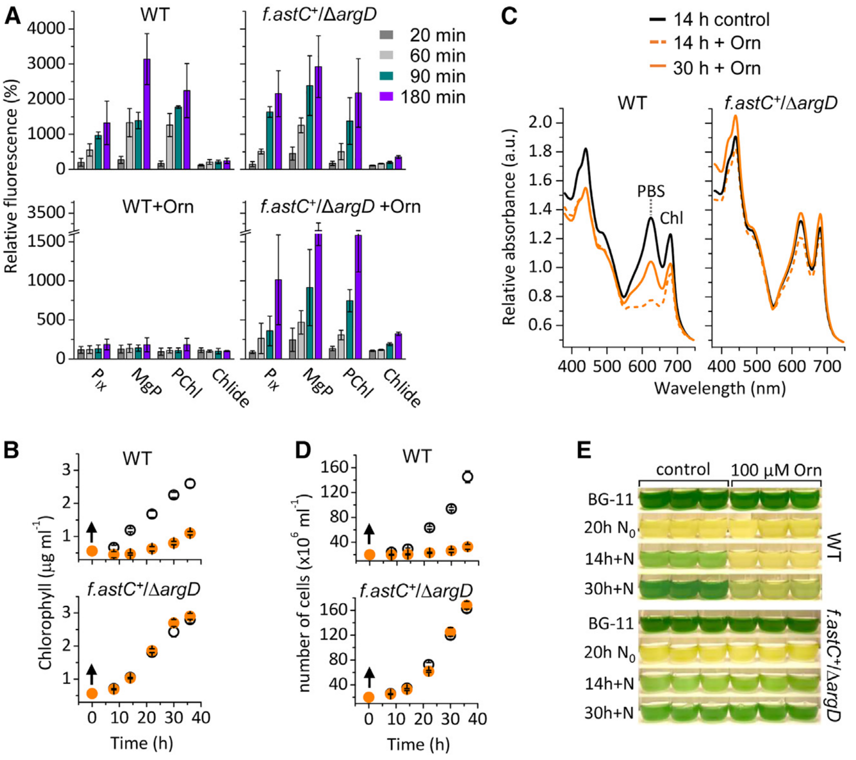

Figure 4. Orn inhibits *de novo* biosynthesis of tetrapyrroles and postpones the recovery of *Synechocystis* WT cells from nitrogen deprivation

(A) The relative amounts of Chl precursors were measured during N repletion of 20 h N-starved cells. The amounts before the addition of 1 mM $NaNO_3$ were taken as 100%.

(B) Changes in Chl content of the cultures after N repletion (indicated by arrows) in the absence (open symbol) or presence (orange symbol) of 100 μM Orn.

(C) *In vivo* cell spectra 14 h after the addition of 1 mM $NaNO_3$ (black line) compared with the spectra measured at 14 (dotted orange line) and 30 h (solid orange line) of N repletion in the presence of 100 μM Orn.

(D) Cell content of the cultures was assessed after the N upshift (indicated by arrows) in the absence (open symbol) or presence (orange symbol) of 100 μM Orn.

(A, B, and D) The averaged data are shown with the error bars representing standard deviation of three independent experiments.

(E) The examined cultures were photographed before (BG-11) and after 20 h of N starvation (N_0) and at indicated times during N repletion (+N) in the absence (control) or presence of 100 μM Orn.

concentration of Gun4 in *Synechocystis* (~1,000 copies per cell) is about half that of ChlH (~2,000 copies per cell).[41] Since this ratio is optimal for the MgCh activity *in vitro*,[20] sequestration of a fraction of Gun4 into soluble complexes could restrict MgP synthesis. In the presence of Orn, we could indeed observe a portion of the total Gun4 redirected from the membrane to the cytoplasm of *f.argD*+/Δ*argD* and WT (Figure 3E). Dependent on the intensity of Chl production in chloroplast, Gun4 was reported to dynamically bind to the ChlH subunit in the membrane or dissociate from it to the soluble fraction.[12,19] We hypothesize that when ArgD *in vivo* binds Gun4 with higher affinity (directly or indirectly triggered by Orn), a population of Gun4, which would move to MgCh, binds ArgD and remains in the cytoplasm. This way, the rate of Chl and bilin production can be modulated by Arg metabolism. *Synechocystis* stores the majority of excess N in its bilin-containing light-harvesting antennae.[32] On the other hand, a considerable amount of the synthesized Arg is directed toward the cyanophycin N stockpile.[1] It is likely that the excess N has to be synchronously biased; therefore, it is conceivable to tune the production of bilins in an Arg biosynthesis-dependent manner via Gun4 acting at the branchpoint of the Chl and heme (bilin) pathways (see Figure 1D). Although not only the product but also the P_{IX} substrate of MgCh were affected (Figures 3A, 4A, and S4A), feedback downregulation of the entire tetrapyrrole biosynthetic pathway has been repeatedly observed for MgCh and Gun4 mutants in plants.[42,43] Also, a number of studies suggest that Gun4 is not merely a protein factor enhancing the activity of MgCh but that it can also directly or indirectly modulate the synthesis of 5-ALA.[44–46]

ArgD is an essential enzyme for the biosynthesis of Orn that is conserved from bacteria to plants. In consequence, an ArgD homolog from *E. coli* (AstC) functionally replaced the *Synechocystis* ArgD in the biosynthesis of Arg (Figures 1A and S1B), but its *in vivo* interaction with Gun4 and CphB was not detectable (Figures 2C and 2D). However, the absence of the Gun4-ArgD interaction did not affect the viability of cells even under severe stress conditions such as fluctuating and/or high light intensities combined with cold stress (Figure S2A), unstable availability of N supply (Figure S2B), or recovery from dormancy (Figures S2C–S2E). These results imply that the presence of this complex is not critical under the tested conditions. Alternatively, Gun4-ArgD might function in the fine regulation of tetrapyrrole biosynthesis, which is difficult to track down unless the intracellular levels of Arg metabolites are intensely altered by Orn supplement (Figure S3A; Table S4). Also, our recent data suggest that there are other sites of cross-talk besides Gun4-ArgD, where the tetrapyrrole and Arg pathways are intertwined in *Synechocystis* (Sobotka, R., unpublished data). Using a similar approach as described here, we discovered a stable protein complex between the bifunctional ArgJ, which catalyzes the first and fifth steps of the Arg biosynthesis (see Figure 1D), and GluTR, a rate-limiting enzyme of 5-ALA formation.[9] Multiple regulatory checkpoints (protein complexes) between these two metabolic pathways can indeed mitigate the absence of a single point, which could explain why the *f.astC*+/Δ*argD* strain showed no defects in viability under the stress conditions tested (Figure S2). Nevertheless, the co-existence of multiple protein-protein interactions further supports the

CellPress
OPEN ACCESS

importance of the co-regulation of Arg metabolism with the biosynthesis of tetrapyrroles.

Limitations of the study

Our working model postulates that at elevated Orn levels, Gun4 preferentially associates with ArgD in the cytoplasm and that its availability for membrane-localized Chl synthesis becomes limited. We observed Gun4 relocalization in Orn-treated cells using immunodetection from fractionated cells (Figure 3E). However, the weakly membrane-bound Gun4[25] could partially dissociate during the preparation of cellular fractions. A better approach would be to monitor the Orn-driven relocalization of Gun4 in intact cells. However, the fluorescent-tagged Gun4 was unstable and prone to degradation. The current study also lacks the identification of a physiological condition in which the discovered ArgD-Gun4 complex plays an important role. This is most likely due to the gap in our knowledge on the eco-physiology of our model strain, which was isolated 52 years ago.[47] Since then, *Synechocystis* has been widely cultivated under optimal laboratory conditions in growth media containing waste amount of nutrients, including N, which particularly undermines studies related to N homeostasis. Consequently, there are no information available on Orn signaling or on the physiological significance of the CphB enzyme in our organism. It conceivably deflects the interpretation of the herein described effect of Orn on the regulation of tetrapyrrole synthesis. Similarly, without knowing the physiological relevance of CphB, it is too early to make conclusion about the importance of its interactions with other proteins in a metabolic network. Therefore, the current report inspires further studies in these directions.

STAR★METHODS

Detailed methods are provided in the online version of this paper and include the following:

- KEY RESOURCES TABLE
- RESOURCE AVAILABILITY
  - Lead contact
  - Materials availability
  - Data and code availability
- EXPERIMENTAL MODEL AND SUBJECT DETAILS
- METHOD DETAILS
  - Construction of the Synechocystis model strains
  - Isolation of Synechocystis soluble and membrane proteins
  - Isolation of protein complexes from Synechocystis
  - Protein electrophoresis, immunoblotting and mass spectrometry
  - Quantification of selected metabolites
  - Purification of recombinant Gun4, CphB and ArgD proteins from Escherichia coli
  - Spectral shift assays
- QUANTIFICATION AND STATISTICAL ANALYSIS

SUPPLEMENTAL INFORMATION

Supplemental information can be found online at https://doi.org/10.1016/j.celrep.2023.113265.

ACKNOWLEDGMENTS

This work was supported by European Research Council Synergy Award 854126.

AUTHOR CONTRIBUTIONS

E.K., J.T., and R.S. designed the study and constructed the strains; E.K., N.B.P.A., S.O., M.M., J.P., T.K., E.S., and P.K. performed the experiments; E.K., M.M., N.B.P.A., P.S., and R.S. analyzed the data; and E.K. and R.S. wrote the paper. All authors read and accepted the final manuscript.

DECLARATION OF INTERESTS

The authors declare no competing interests.

Received: February 14, 2023
Revised: August 11, 2023
Accepted: September 29, 2023
Published: October 20, 2023

REFERENCES

1. Zhang, H., Liu, Y., Nie, X., Liu, L., Hua, Q., Zhao, G.P., and Yang, C. (2018). The cyanobacterial ornithine-ammonia cycle involves an arginine dihydrolase. Nat. Chem. Biol. *14*, 575–581. https://doi.org/10.1038/s41589-018-0038-z.
2. Watzer, B., and Forchhammer, K. (2018). Cyanophycin synthesis optimizes nitrogen utilization in the unicellular cyanobacterium *Synechocystis* sp. strain PCC 6803. Appl. Environ. Microbiol. *84*, e01298-18–e01218. https://doi.org/10.1128/AEM.01298-18.
3. Görl, M., Sauer, J., Baier, T., and Forchhammer, K. (1998). Nitrogen-starvation-induced chlorosis in *Synechococcus* PCC 7942: adaptation to long-term survival. Microbiology *144*, 2449–2458, (Pt 9). https://doi.org/10.1099/00221287-144-9-2449.
4. Wase, N., Black, P.N., Stanley, B.A., and DiRusso, C.C. (2014). Integrated quantitative analysis of nitrogen stress response in *Chlamydomonas reinhardtii* using metabolite and protein profiling. J. Proteome Res. *13*, 1373–1396. https://doi.org/10.1021/pr400952z.
5. Klotz, A., Georg, J., Bučinská, L., Watanabe, S., Reimann, V., Januszewski, W., Sobotka, R., Jendrossek, D., Hess, W.R., and Forchhammer, K. (2016). Awakening of a dormant cyanobacterium from nitrogen chlorosis reveals a genetically determined program. Curr. Biol. *26*, 2862–2872. https://doi.org/10.1016/j.cub.2016.08.054.
6. Brzezowski, P., Richter, A.S., and Grimm, B. (2015). Regulation and function of tetrapyrrole biosynthesis in plants and algae. Biochim. Biophys. Acta *1847*, 968–985. https://doi.org/10.1016/j.bbabio.2015.05.007.
7. Papenbrock, J., Mishra, S., Mock, H.P., Kruse, E., Schmidt, E.K., Petersmann, A., Braun, H.P., and Grimm, B. (2001). Impaired expression of the plastidic ferrochelatase by antisense RNA synthesis leads to a necrotic phenotype of transformed tobacco plants. Plant J. *28*, 41–50. https://doi.org/10.1046/j.1365-313x.2001.01126.x.
8. Vavilin, D., Brune, D.C., and Vermaas, W. (2005). N-15-labeling to determine chlorophyll synthesis and degradation in *Synechocystis* sp. PCC 6803 strains lacking one or both photosystems. Biochim. Biophys. Acta *1708*, 91–101. https://doi.org/10.1016/j.bbabio.2004.12.011.
9. Wang, P., Ji, S., and Grimm, B. (2022). Post-translational regulation of metabolic checkpoints in plant tetrapyrrole biosynthesis. J. Exp. Bot. *73*, 4624–4636, 10.1093/jxb/erac203. https://doi.org/10.1093/jxb/erac1203.
10. Davison, P.A., Schubert, H.L., Reid, J.D., Iorg, C.D., Heroux, A., Hill, C.P., and Hunter, C.N. (2005). Structural and biochemical characterization of Gun4 suggests a mechanism for its role in chlorophyll biosynthesis. Biochemistry *44*, 7603–7612. https://doi.org/10.1021/bi050240x.
11. Verdecia, M.A., Larkin, R.M., Ferrer, J.L., Riek, R., Chory, J., and Noel, J.P. (2005). Structure of the Mg-chelatase cofactor GUN4 reveals a novel

hand-shaped fold for porphyrin binding. PLoS Biol. *3*, e151. https://doi.org/10.1371/journal.pbio.0030151.

12. Adhikari, N.D., Orler, R., Chory, J., Froehlich, J.E., and Larkin, R.M. (2009). Porphyrins promote the association of GENOMES UNCOUPLED 4 and a Mg-chelatase subunit with chloroplast membranes. J. Biol. Chem. *284*, 24783–24796. https://doi.org/10.1074/jbc.M109.025205.
13. Chen, X., Pu, H., Wang, X., Long, W., Lin, R., and Liu, L. (2015). Crystal Structures of GUN4 in Complex with Porphyrins. Mol. Plant *8*, 1125–1127. https://doi.org/10.1016/j.molp.2015.04.013.
14. Adams, N.B.P., Brindley, A.A., Hunter, C.N., and Reid, J.D. (2016). The catalytic power of magnesium chelatase: a benchmark for the AAA+ ATPases. FEBS Lett. *590*, 1687–1693. https://doi.org/10.1002/1873-3468.12214.
15. Hu, J.H., Chang, J.W., Xu, T., Wang, J., Wang, X., Lin, R., Duanmu, D., and Liu, L. (2021). Structural basis of bilin binding by the chlorophyll biosynthesis regulator GUN4. Protein Sci. *30*, 2083–2091. https://doi.org/10.1002/pro.4164.
16. Zhang, W., Willows, R.D., Deng, R., Li, Z., Li, M., Wang, Y., Guo, Y., Shi, W., Fan, Q., Martin, S.S., et al. (2021). Bilin-dependent regulation of chlorophyll biosynthesis by GUN4. Proc. Natl. Acad. Sci. USA *118*, e2104443118. https://doi.org/10.1073/pnas.2104443118.
17. Fölsche, V., Großmann, C., and Richter, A.S. (2022). Impact of porphyrin binding to GENOMES UNCOUPLED 4 on tetrapyrrole biosynthesis in planta. Front. Plant Sci. *13*, 850504. https://doi.org/10.3389/fpls.2022.850504.
18. Wilde, A., Mikolajczyk, S., Alawady, A., Lokstein, H., and Grimm, B. (2004). The *gun4* gene is essential for cyanobacterial porphyrin metabolism. FEBS Lett. *571*, 119–123. https://doi.org/10.1016/j.febslet.2004.06.063.
19. Adhikari, N.D., Froehlich, J.E., Strand, D.D., Buck, S.M., Kramer, D.M., and Larkin, R.M. (2011). GUN4-porphyrin complexes bind the ChlH/GUN5 subunit of Mg-chelatase and promote chlorophyll biosynthesis in Arabidopsis. Plant Cell *23*, 1449–1467. https://doi.org/10.1105/tpc.110.082503.
20. Kopečná, J., Cabeza de Vaca, I., Adams, N.B.P., Davison, P.A., Brindley, A.A., Hunter, C.N., Guallar, V., and Sobotka, R. (2015). Porphyrin binding to Gun4 protein, facilitated by a flexible loop, controls metabolite flow through the chlorophyll biosynthetic pathway. J. Biol. Chem. *290*, 28477–28488. https://doi.org/10.1074/jbc.M115.664987.
21. Formighieri, C., Ceol, M., Bonente, G., Rochaix, J.D., and Bassi, R. (2012). Retrograde signaling and photoprotection in a *gun4* mutant of *Chlamydomonas reinhardtii*. Mol. Plant *5*, 1242–1262. https://doi.org/10.1093/mp/sss051.
22. Li, R.-Q., Jiang, M., Liu, Y.-H., Zheng, Y.-C., Huang, J.-Z., Wu, J.-M., and Shu, Q.-Y. (2017). The xantha marker trait is associated with altered tetrapyrrole biosynthesis and deregulated transcription of PhANGs in rice. Front. Plant Sci. *8*, 901. https://doi.org/10.3389/fpls.2017.00901.
23. Mochizuki, N., Tanaka, R., Tanaka, A., Masuda, T., and Nagatani, A. (2008). The steady-state level of Mg-protoporphyrin IX is not a determinant of plastid-to-nucleus signaling in Arabidopsis. Proc. Natl. Acad. Sci. USA *105*, 15184–15189. https://doi.org/10.1073/pnas.0803245105.
24. Larkin, R.M., Alonso, J.M., Ecker, J.R., and Chory, J. (2003). GUN4, a regulator of chlorophyll synthesis and intracellular signaling. Science *299*, 902–906. https://doi.org/10.1126/science.1079978.
25. Sobotka, R., Dühring, U., Komenda, J., Peter, E., Gardian, Z., Tichý, M., Grimm, B., and Wilde, A. (2008). Importance of the cyanobacterial GUN4 protein for chlorophyll metabolism and assembly of photosynthetic complexes. J. Biol. Chem. *283*, 25794–25802. https://doi.org/10.1074/jbc.M803787200.
26. Richter, R., Hejazi, M., Kraft, R., Ziegler, K., and Lockau, W. (1999). Cyanophycinase, a peptidase degrading the cyanobacterial reserve material multi-L-arginyl-poly-L-aspartic acid (cyanophycin): molecular cloning of the gene of *Synechocystis* sp. PCC 6803, expression in *Escherichia coli*, and biochemical characterization of the purified enzyme. Eur. J. Biochem. *263*, 163–169. https://doi.org/10.1046/j.1432-1327.1999.00479.x.
27. Li, H., Sherman, D.M., Bao, S., and Sherman, L.A. (2001). Pattern of cyanophycin accumulation in nitrogen-fixing and non-nitrogen-fixing cyanobacteria. Arch. Microbiol. *176*, 9–18. https://doi.org/10.1007/s002030100281.
28. Quintero, M.J., Muro-Pastor, A.M., Herrero, A., and Flores, E. (2000). Arginine catabolism in the cyanobacterium *Synechocystis* sp. strain PCC 6803 involves the urea cycle and arginase pathway. J. Bacteriol. *182*, 1008–1015. https://doi.org/10.1128/jb.182.4.1008-1015.2000.
29. Flores, E., Arévalo, S., and Burnat, M. (2019). Cyanophycin and arginine metabolism in cyanobacteria. Algal Res. *42*, 101577. https://doi.org/10.1016/j.algal.2019.101577.
30. Sobotka, R. (2014). Making proteins green; biosynthesis of chlorophyll-binding proteins in cyanobacteria. Photosynth. Res. *119*, 223–232. https://doi.org/10.1007/s11120-013-9797-2.
31. Koskela, M.M., Skotnicová, P., Kiss, É., and Sobotka, R. (2020). Purification of protein-complexes from the cyanobacterium *Synechocystis* sp. PCC 6803 using FLAG-affinity chromatography. Bio. Protoc. *10*, e3616. https://doi.org/10.21769/BioProtoc.3616.
32. Li, Z.-M., Bai, F., Wang, X., Xie, C., Wan, Y., Li, Y., Liu, J., and Li, Z. (2023). Kinetic characterization and catalytic mechanism of N-acetylornithine aminotransferase encoded by *slr1022* gene from *Synechocystis* sp. PCC 6803. Int. J. Mol. Sci. *24*, 5853. https://doi.org/10.3390/ijms24065853.
33. Baers, L.L., Breckels, L.M., Mills, L.A., Gatto, L., Deery, M.J., Stevens, T.J., Howe, C.J., Lilley, K.S., and Lea-Smith, D.J. (2019). Proteome mapping of a cyanobacterium reveals distinct compartment organization and cell-dispersed metabolism. Plant Physiol. *181*, 1721–1738. https://doi.org/10.1104/pp.19.00897.
34. Quintero, M.J., Montesinos, M.L., Herrero, A., and Flores, E. (2001). Identification of genes encoding amino acid permeases by inactivation of selected ORFs from the Synechocystis genomic sequence. Genome Res. *11*, 2034–2040. https://doi.org/10.1101/gr.196301.
35. Cunin, R., Glansdorff, N., Piérard, A., and Stalon, V. (1986). Biosynthesis and metabolism of arginine in bacteria. Microbiol. Rev. *50*, 314–352. https://doi.org/10.1128/mr.50.3.314-352.1986.
36. Abadjieva, A., Pauwels, K., Hilven, P., and Crabeel, M. (2001). A new yeast metabolon involving at least the two first enzymes of arginine biosynthesis: acetylglutamate synthase activity requires complex formation with acetylglutamate kinase. J. Biol. Chem. *276*, 42869–42880. https://doi.org/10.1074/jbc.M103732200.
37. Chen, G.E., Hitchcock, A., Mareš, J., Gong, Y., Tichý, M., Pilný, J., Kovářová, L., Zdvihalová, B., Xu, J., Hunter, C.N., and Sobotka, R. (2021). Evolution of Ycf54-independent chlorophyll biosynthesis in cyanobacteria. Proc. Natl. Acad. Sci. USA *118*, e2024633118. https://doi.org/10.1073/pnas.2024633118.
38. Schriek, S., Rückert, C., Staiger, D., Pistorius, E.K., and Michel, K.-P. (2007). Bioinformatic evaluation of L-arginine catabolic pathways in 24 cyanobacteria and transcriptional analysis of genes encoding enzymes of L-arginine catabolism in the cyanobacterium *Synechocystis* sp. PCC 6803. BMC Genom. *8*, 437. https://doi.org/10.1186/1471-2164-8-437.
39. Xiong, W., Brune, D., and Vermaas, W.F.J. (2014). The γ-aminobutyric acid shunt contributes to closing the tricarboxylic acid cycle in *Synechocystis* sp. PCC 6803. Mol. Microbiol. *93*, 786–796. https://doi.org/10.1111/mmi.12699.
40. Adams, N.B.P., Marklew, C.J., Qian, P., Brindley, A.A., Davison, P.A., Bullough, P.A., and Hunter, C.N. (2014). Structural and functional consequences of removing the N-terminal domain from the magnesium chelatase ChlH subunit of *Thermosynechococcus elongatus*. Biochem. J. *464*, 315–322. https://doi.org/10.1042/bj20140463.
41. Jackson, P.J., Hitchcock, A., Brindley, A.A., Dickman, M.J., and Hunter, C.N. (2023). Absolute quantification of cellular levels of photosynthesis-related proteins in *Synechocystis* sp. PCC 6803. Photosynth. Res. *155*, 219–245. https://doi.org/10.1007/s11120-022-00990-z.

CellPress
OPEN ACCESS

42. Papenbrock, J., Mock, H.P., Tanaka, R., Kruse, E., and Grimm, B. (2000). Role of magnesium chelatase activity in the early steps of the tetrapyrrole biosynthetic pathway. Plant Physiol. *122*, 1161–1169. https://doi.org/10.1104/pp.122.4.1161.

43. Richter, A.S., Hochheuser, C., Fufezan, C., Heinze, L., Kuhnert, F., and Grimm, B. (2016). Phosphorylation of GENOMES UNCOUPLED 4 alters stimulation of Mg chelatase activity in angiosperms. Plant Physiol. *172*, 1578–1595. https://doi.org/10.1104/pp.16.01036.

44. Peter, E., and Grimm, B. (2009). GUN4 is required for posttranslational control of plant tetrapyrrole biosynthesis. Mol. Plant *2*, 1198–1210. https://doi.org/10.1093/mp/ssp072.

45. Brzezowski, P., Schlicke, H., Richter, A., Dent, R.M., Niyogi, K.K., and Grimm, B. (2014). The GUN4 protein plays a regulatory role in tetrapyrrole biosynthesis and chloroplast-to-nucleus signalling in *Chlamydomonas reinhardtii*. Plant J. *79*, 285–298. https://doi.org/10.1111/tpj.12560.

46. Wang, P., Richter, A.S., Kleeberg, J.R.W., Geimer, S., and Grimm, B. (2020). Post-translational coordination of chlorophyll biosynthesis and breakdown by BCMs maintains chlorophyll homeostasis during leaf development. Nat. Commun. *11*, 1254. https://doi.org/10.1038/s41467-020-14992-9.

47. Stanier, R.Y., Kunisawa, R., Mandel, M., and Cohen-Bazire, G. (1971). Purification and properties of unicellular blue-green algae (order Chroococcales). Bacteriol. Rev. *35*, 171–205. https://doi.org/10.1128/br.35.2.171-205.1971.

48. Tichý, M., Bečková, M., Kopečná, J., Noda, J., Sobotka, R., and Komenda, J. (2016). Strain of *Synechocystis* PCC 6803 with aberrant assembly of photosystem II contains tandem duplication of a large chromosomal region. Front. Plant Sci. *7*, 648. https://doi.org/10.3389/fpls.2016.00648.

49. Chidgey, J.W., Linhartová, M., Komenda, J., Jackson, P.J., Dickman, M.J., Canniffe, D.P., Koník, P., Pilný, J., Hunter, C.N., and Sobotka, R. (2014). A cyanobacterial chlorophyll synthase-HliD complex associates with the Ycf39 protein and the YidC/Alb3 insertase. Plant Cell *26*, 1267–1279. https://doi.org/10.1105/tpc.114.124495.

50. Schneider, C.A., Rasband, W.S., and Eliceiri, K.W. (2012). NIH Image to ImageJ: 25 years of image analysis. Nat Meth *9*, 671–675. https://doi.org/10.1038/nmeth.2089.

51. Spät, P., Klotz, A., Rexroth, S., Maček, B., and Forchhammer, K. (2018). Chlorosis as a developmental program in cyanobacteria: the proteomic fundament for survival and awakening. Mol. Cell. Proteomics *17*, 1650–1669. https://doi.org/10.1074/mcp.RA118.000699.

52. Hollingshead, S., Kopecná, J., Jackson, P.J., Canniffe, D.P., Davison, P.A., Dickman, M.J., Sobotka, R., and Hunter, C.N. (2012). Conserved chloroplast open-reading frame ycf54 is required for activity of the magnesium protoporphyrin monomethylester oxidative cyclase in *Synechocystis* PCC 6803. J. Biol. Chem. *287*, 27823–27833. https://doi.org/10.1074/jbc.M112.352526.

53. Kiss, É., Knoppová, J., Aznar, G.P., Pilný, J., Yu, J., Halada, P., Nixon, P.J., Sobotka, R., and Komenda, J. (2019). A photosynthesis-specific rubredoxin-like protein is required for efficient association of the D1 and D2 proteins during the initial steps of photosystem II assembly. Plant Cell *31*, 2241–2258. https://doi.org/10.1105/tpc.19.00155.

54. Komenda, J. (2005). Autotrophic cells of the Synechocystis *psbH* deletion mutant are deficient in synthesis of CP47 and accumulate inactive PSII core complexes. Photosynth. Res. *85*, 161–167. https://doi.org/10.1007/s11120-005-1294-9.

55. Bučinská, L., Kiss, É., Konik, P., Knoppová, J., Komenda, J., and Sobotka, R. (2018). The ribosome-bound protein Pam68 promotes insertion of chlorophyll into the CP47 subunit of Photosystem II. Plant Physiol *4*, 2931–2942. https://doi.org/10.1104/pp.18.00061.

56. Pilný, J., Kopečná, J., Noda, J., and Sobotka, R. (2015). Detection and quantification of heme and chlorophyll precursors using a High Performance Liquid Chromatography (HPLC) system equipped with two fluorescence detectors. Bio-protocol *5*, e1390. https://doi.org/10.21769/BioProtoc.1390.

57. Ritchie, R.J. (2006). Consistent sets of spectrophotometric chlorophyll equations for acetone, methanol and ethanol solvents. Photosynth. Res. *89*, 27–41. https://doi.org/10.1007/s11120-006-9065-9.

58. Opekar, S., Kvíčala, J., Moos, M., Pejchal, V., and Šimek, P. (2021). Mechanism of alkyl chloroformate-mediated esterification of carboxylic acids in aqueous media. J. Org. Chem. *86*, 16293–16299. https://doi.org/10.1021/acs.joc.1c01546.

59. Langer, A., Bartoschik, T., Cehlar, O., Duhr, S., Baaske, P., and Streicher, W. (2022). A new spectral shift-based method to characterize molecular interactions. Assay Drug Dev. Technol. *20*, 83–94. https://doi.org/10.1089/adt.2021.133.

STAR★METHODS

KEY RESOURCES TABLE

REAGENT or RESOURCE	SOURCE	IDENTIFIER
Antibodies		
anti-FLAG	Sigma-Aldrich	Cat# F7425, RRID:AB_439687
anti-Gun4	Raised in rabbit against the recombinant *Synechocystis* Gun4 (Sobotka et al.[25])	N/A
anti-CphB	Raised in rabbit against the recombinant *Synechocystis* CphB (this study).	N/A
anti-ArgD	Raised in rabbit against the recombinant *Synechocystis* ArgD (this study).	N/A
anti-rabbit IgG-peroxidase antibody produced in goat	Sigma-Aldrich	Cat# A6154, RRID:AB_11125345
goat-*anti*-rabbit IgG IR800	Azure Biosystems	Cat# AC2134
Bacterial strains		
WT-P substrain of *Synechocystis* sp. PCC 6803 (used as a background for all other strains of this study).	Tichý et al.[48]	N/A
Δ*argD Synechocystis* sp. PCC 6803	This study	N/A
f.argD$^+$/Δ*argD Synechocystis* sp. PCC 6803	This study	N/A
f.astC$^+$/Δ*argD Synechocystis* sp. PCC 6803	This study	N/A
Δ*cphB Synechocystis* sp. PCC 6803	This study	N/A
f.argD$^+$/Δ*cphB Synechocystis* sp. PCC 6803	This study	N/A
f.gun4$^+$/*Δgun4 Synechocystis* sp. PCC 6803	Sobotka et al.[25]	N/A
pET21a-gun4 Escherichia coli	This study	N/A
pET21a-argD Escherichia coli	This study	N/A
pET21a-cphB Escherichia coli	This study	N/A
Chemicals, peptides, and recombinant proteins		
anti-FLAG M2 affinity gel	Sigma-Aldrich	Cat# A2220
3xFLAG peptide	Sigma-Aldrich	Cat# A4799
Gun4-6xHis	This study	N/A
ArgD-6xHis	This study	N/A
CphB-6xHis	This study	N/A
Deposited data		
Proteomics data (identification of protein gel bands)	MassIVE: https://massive.ucsd.edu/ProteoSAFe/static/massive.jsp	MassIVE: MSV000091280
Metabolomics data	figshare: https://figshare.com/	figshare: https://doi.org/10.6084/m9.figshare.24205368.v1
Oligonucleotides		
For list of primers see Supplementary Table S5	This study	N/A
Recombinant DNA		
pPD-*N*FLAG (Km)	Chidgey et al.[49]	N/A
Software and algorithms		
Thermo Xcalibur	Thermo Fisher Scientific	RRID:SCR_014593
MassHunter Quantitative Analysis software	Agilent	RRID:SCR_015040
ImageJ	Schneider et al.[50]	RRID:SCR_003070

RESOURCE AVAILABILITY

Lead contact

Further information and requests for resources and reagents should be directed to and will be fulfilled by the lead contact, Roman Sobotka (sobotka@alga.cz).

Materials availability

The *E. coli* and *Synechocystis* strains generated in this study will be sent after request from the lead contact.

Data and code availability

- Proteomics data have been deposited at MassIVE: MSV000091280 and are publicly available as of the date of publication. Accession numbers are listed in the key resources table. Metabolomics data have been deposited at figshare (https://doi.org/10.6084/m9.figshare.24205368.v1) and are publicly available as of the date of publication.
- Further data reported in this paper, and any additional information required to reanalyze the data reported in this paper will be shared by the lead contact upon request.
- This paper does not report original code.

EXPERIMENTAL MODEL AND SUBJECT DETAILS

Synechocystis sp. PCC 6803 substrain GT-P[48] was used as the wild type (WT) and as a genetic background for all prepared strains listed in Table S1. Unless stated otherwise, the *Synechocystis* strains were grown photoautotrophically in liquid BG-11 medium on a rotary shaker at 28°C, under continuous, moderate irradiance of 40 μmol photons m^{-2} s^{-1} given by white fluorescence tubes. The plate-drop experiments were performed by pipetting liquid cultures on a BG-11 agar plate. The drops were photographed after three days of photoautotrophic growth at 30°C, under constant illumination with 30 μmol photons m^{-2} s^{-1}. Alternatively, the plates were exposed to 500 μmol photons m^{-2} s^{-1} (high light) at 28 or 22°C; or to repeated periods of 5 min illumination with high light and 5 min dark (fluctuating light). The fluctuating nitrogen (N) stress was mimicked by repeated cycles of washing the cells to nitrate-less BG-11 (N_0) for 8 h; followed by the addition of 18 mM $NaNO_3$ supplement to the liquid cultures for another 8 h; while agitated with 240 rpm at 28°C, illuminated by 200 μmol m^{-2} s^{-1}. Dormancy and recovery were induced by 25 d of N-starvation[51] and the addition of 1 mM $NaNO_3$, respectively. When amino acids were included to the media the stable pH was ensured by 10 mM TES. The absorption spectra of cells were measured by a UV-3000 spectrophotometer (Shimadzu). The number and average size of cells were assessed by coulter counter (Multisizer 4, Beckman Coulter).

METHOD DETAILS

Construction of the Synechocystis model strains

The Δ*argD* and Δ*cphB* strains were constructed by replacing the *slr1022* and *slr2001* genes by erythromycin or spectinomycin resistance cassettes, respectively. The primers in the megaprimer PCR method used for mutagenesis are listed in Table S5. The segregation of Δ*argD* locus was achieved in the presence of 5 mM Orn. For the construction of strains expressing the N-terminally 3xFLAG-tagged ArgD protein (f.ArgD) we purchased a synthetic *Synechocystis argD* gene (GenScript, USA) with an optimized codon usage to remove the common restriction binding sites. To express the AstC enzyme from *Escherichia coli* (*E. coli*) in *Synechocystis*, the *astC* gene of *E. coli* was amplified by PCR from the genomic DNA. The *argD* and *astC* genes were cloned to the pPD-*N*FLAG plasmid[52,49] (for all primers used see Table S5). The obtained constructs were transformed into *Synechocystis* Δ*argD* and/or Δ*cphB* cells. All transformed *Synechocystis* cells were fully segregated on BG-11 plates with increasing concentrations of relevant antibiotics (see Table S1).

Isolation of Synechocystis soluble and membrane proteins

Synechocystis cells (5 x 10^9) from exponential growth phase were pelleted, washed, and re-suspended in buffer A containing 20 mM HEPES pH 7.4, 5 mM $CaCl_2$, 10 mM $MgCl_2$, 25% glycerol, protease inhibitor (cOmplete; Roche). The cells were broken mechanically in a Precellys Evolution tissue homogenizer (Bertin Instruments) using balotina beads (100–200 μm). The breaking was performed in three cycles of shaking at 7500 rpm at 0°C; the sample was chilled for 2 min between the cycles. After removing the beads by repeated washing and sedimentation steps, the insoluble and soluble fractions were separated by three series of centrifugation at high speed (4°C, 65000 *x* ***g***, 20 min).

Isolation of protein complexes from Synechocystis

Cells from 4 L of *Synechocystis* cultures (*c*. 2x10^8 mL^{-1} cell content) were centrifuged, washed with buffer A and broken as described in the 'Isolation of *Synechocystis* soluble proteins' section. The soluble fraction was applied to anti-FLAG M2 affinity gel chromatography (Sigma-Aldrich) essentially as described in.[31] Proteins bound to the column were washed with 15 column volumes of buffer A. FLAG-tagged protein complexes were eluted with synthetic 3xFLAG peptide (150 μg/mL) in buffer A. Alternatively, the proteins were eluted form the column with 1% SDS in buffer A.

Protein electrophoresis, immunoblotting and mass spectrometry

The isolated proteins and protein complexes were solubilized and separated on SDS-PAGE essentially as described in[53]; and subsequently stained by Coomassie Blue. In case of immunoblot, the gel was stained by SYPRO Orange (Sigma-Aldrich) and

transferred onto a PVDF membrane that was subsequently incubated with specific primary antibody and then with secondary antibody conjugated with horseradish peroxidase (Sigma-Aldrich). The following primary antibodies were used in the study: anti-Gun4,[25] anti-D1[54]; anti-FLAG (Sigma-Aldrich Cat# F7425, RRID:AB_439687); the polyclonal anti-CphB and anti-ArgD antibodies were generated in rabbit, against the full-length *Synechocystis* recombinant proteins produced in *E. coli* (Moravia Biotech). The primary antibodies were probed with anti-rabbit IgG-peroxidase antibody produced in goat (Sigma-Aldrich Cat# A6154, RRID:AB_11125345) and visualized using Immobilon Crescendo Western HRP substrate (Millipore, Cat# WBLUR0500, RRID:AB_439687) and luminescence image analyzer (ImageQuant, LAS-4000). Alternatively, we used goat-*anti*-rabbit IgG IR800 antibody (Cat# AC2134) and visualize the signal using Azure Biosystems. For the identification of proteins by liquid chromatography (LC) coupled tandem mass spectrometry (MS/MS), the Coomassie Blue-stained bands were cut from the gel, digested and analyzed as described here.[55] For protein identification, MS/MS spectra were searched against *Synechocystis* species-specific protein bases (UNIPROT Universal Protein Resource, (RRID:SCR_002380) and CyanoBase (RRID:SCR_007615)) using the PLGS3.0 (Waters) software package.

Quantification of selected metabolites

The biosynthetic precursors of Chl/heme were extracted from equal number of cells and quantified by HPLC, essentially as described in.[56] The Chl content per cell was determined for three independent cultures after methanol extraction of pigments according to.[57] For the quantification of selected metabolites by combined GC-MS and LC-MS analysis[58] 3×10^9 cells were collected by centrifugation and frozen in liquid N. Cell pellets were dried and immediately extracted with 400 μL of a cold extraction medium methanol:ACN:H2O (2:2:1 v/v/v) containing an internal standard 4-fluorophenylalanine (80 nmol). The sample was then homogenized using a Tissue Lyser II (Qiagen) at 50 Hz, 0°C for 5 min. The mixture was then centrifuged at (8000 ***g***, 10 min, 5°C). The supernatant was removed, and the extraction step was repeated under the same conditions but without the internal standard. The supernatants were combined and the obtained sample extract stored (−80°C). A 100 μL aliquot of each extract was mixed with ^{13}C-labeled internal standards ($^{13}C_3$-Serine, $^{13}C_3$-Alanine, $^{13}C_6$-Tyrosine, $^{13}C_2$-Glutamic acid, $^{13}C_6$-Arginine, $^{13}C_6$-Lysine, $^{13}C_6$-Phenylalanine, $^{13}C_4$-Asparagine, $^{13}C_5$-Methionine, $^{13}C_2$-Threonine, $^{13}C_4$-2-Oxoglutarate, $^{13}C_5$-Glutamine, $^{13}C_5$-Proline, $^{13}C_6$-Arginine) (absolute 2 nmol each) were concentrated in a vacuum concentrator (RVC 2–25 CD Plus combined with ALPHA 1–2 LD Plus, Thermo-Fischer Scientific). Each dried extract sample was then subjected to derivatization with ethanol (EtOH) - ethyl chloroformate (ECF) reaction medium under pyridine catalysis and simultaneous liquid-liquid microextraction into a lower chloroform layer as described earlier.[58] Briefly, the following five sequential steps were used for the addition of a corresponding medium to the evaporated sample extract: (1) 50 μL of the mixture EtOH: water (2:1; v/v), (2) 50 μL of the mixture EtOH: pyridine (2:1; v/v), (3) 50 μL of the mixture ECF: chloroform (1:7; v/v), (4) 50 μL 1 M NaOH, (5) 50 μL of the mixture ECF: chloroform (1:7; v/v). The reaction mixture was stirred before addition of the particular medium. Finally, 30 μL of the lower chloroform layer was evaporated by a gentle stream of nitrogen and redissolved in 100 μL methanol: water (3:7; v/v) for LC-MS analysis. 50 μL of 1 M HCl was added to the remaining reaction mixture and stirred. The 50 μL of the lower chloroform phase was used for GC-MS analysis. An LTQ XL mass spectrometer coupled to a Accela 600 liquid chromatograph (LC) and a Accela autosampler (all Thermo Fisher Scientific) was used for quantitative analysis. Amino acids were separated on a 150 mm × 3 mm i.d., 2.6 μm, Kinetex C18 (Phenomenex) with a mobile phase flow rate of 400 μL/min, an injection volume of 5 μL, and a column temperature of 35°C. The mobile phase was A = 5 mmol/L ammonium format in methanol, B = 5 mmol/L aqueous ammonium format; gradient (A): 0.0 min, 30%; 10.0 min, 100%; 11.0 min, 100%; 11.1 min, 30%; 14.5 min, 30%. Full scan positive ion mass spectra were acquired in a mass range of 85–850 Da. LT-Q settings were as follows: 2.5 kV spray voltage ion source parameter, 300°C capillary temperature, sheath gas at 40 au, aux gas at 10 au, spare gas at 1 au, 300°C source temperature. Data were processed using Thermo Xcalibur software (Thermo Fisher Scientific, RRID:SCR_014593), version 4.0. GC-MS analyses of amino acids were performed using a VF-17ms capillary column (30 m, 250 mm, 250 μm) and a gas chromatograph 5977B coupled to a quadrupole mass spectrometer 5977B MSD (Agilent) equipped with an electron ionization source (EI) and operated in full-scan mode (40–500 Da). The instrument settings were: Helium flow rate, 1.2 mL/min; inlet temperature, 280°C; injection mode, splitless; split flow, 40 mL/min; splitless time, 1.0 min; septum purge flow 3 mL/min; temperature program, 45°C, hold for 2 min, 16°C/min to 320°C, hold for 2 min; transfer line temperature, 280°C; and EI source temperature, 230°C; ionization energy, 70 eV. Data were processed using Agilent MassHunter WorkStation - Qualitative Analysis for GC/MS (Agilent, RRID:SCR_016657) and Agilent Masshunter Quantitative Analysis software (Agilent, RRID:SCR_015040) version 10.

Purification of recombinant Gun4, CphB and ArgD proteins from Escherichia coli

C-terminal His6-tagged Gun4, CphB and ArgD proteins were over-expressed in *E. coli* BL21 (DE3) using a pET21a plasmid (Novagen). The expression was induced with isopropyl-β-D-thiogalactopyranoside (0.4 mM) and shaken for additional 20 h at 18°C. Cells were harvested by centrifugation (10 min, 4°C, 10000 × ***g***), resuspended in lysis buffer (25 mM Tris/HCl pH 7.8, 150 mM NaCl, 10 mg L^{-1} of DNase I, 10 mg L^{-1} of lysozyme, protease inhibitor (cOmplete; Roche), and incubated for 30 min at 37°C. After incubation, cells were disrupted by sonication and the lysate was clarified by centrifugation (4°C, 30 min, 10000 × ***g***). Soluble His-tagged proteins were purified by metal-affinity chromatography (Protino Ni-NTA agarose, Macherey-Nagel; buffer: 25 mM Tris/HCl pH 8.0, 150 mM NaCl); their purity and concentration were checked by SDS-PAGE.

Spectral shift assays

Recombinant Gun4 and ArgD proteins were labeled with 2nd Generation Red NHS dye using standard protocols (NanoTemper Technologies GmbH, Munich, Germany) and used for isothermal spectral shift assays.[59] 5 or 20 nM labeled ArgD or Gun4 was titrated with a serial dilution of CphB or Gun4, respectively. Binding experiment were performed in triplicate, in 25 mM Tris, 0.1% Pluronic F-127, pH 8 at 25°C. Samples were loaded into Monolith Premium capillaries (NanoTemper Technologies) and loaded into a Monolith X instrument (NanoTemper Technologies) and excited at 590 nm (100% LED power), using software version Mo.Control 2.4.1. The ratio of fluorescence emission at 670 : 650 nm was collected; and the data were exported to CSV files, where custom Python scripts were used to determine the dissociation constant (K_d) using the equations in.[59]

QUANTIFICATION AND STATISTICAL ANALYSIS

The ratios of the co-purified f.ArgD and Gun4 proteins indicated on Figures 3B and 3C were assessed from the intensity of the stained protein bands or antibody signals using the ImageJ software (RRID:SCR_003070).[50] The average (arithmetic mean) and standard deviation of the data (shown on Figures 3A and 4A, 4B and 4D; S4B and S4E) were determined from measurements of n = 3 samples. Significance of the data (where indicated) was tested with one-tailed *t* test, with a significance level set to $p < 0.05$.

Cell Reports, Volume *42*

Supplemental information

Chlorophyll biosynthesis

under the control of arginine metabolism

Éva Kiss, Jana Talbot, Nathan B.P. Adams, Stanislav Opekar, Martin Moos, Jan Pilný, Tatjana Kvasov, Emilia Schneider, Peter Koník, Petr Šimek, and Roman Sobotka

Supplemental information for:

Chlorophyll biosynthesis under the control of arginine metabolism

Éva Kiss[1], Jana Talbot[1#], Nathan B.P. Adams[2,3], Stanislav Opekar[4], Martin Moos[4], Jan Pilný[1], Tatjana Kvasov[2], Emilia Schneider[2], Peter Koník[1,5], Petr Šimek[4], Roman Sobotka[1,5*]

[1]Laboratory of Photosynthesis, Centre Algatech, Institute of Microbiology, Czech Academy of Sciences, 37901 Třeboň, Czech Republic; [2]NanoTemper Technologies, Floessegasse 4, Munich, 81369, Germany; [3]Department of Molecular Biology and Biotechnology, University of Sheffield, Sheffield S10 2TN, United Kingdom; [4]Biology Centre of the Czech Academy of Sciences, Branišovská 1160/31, 370 05 České Budějovice, Czech Republic; [5]Faculty of Science, University of South Bohemia, 37005 České Budějovice, Czech Republic

[#]Present address: Wicking Dementia Research and Education Centre, University of Tasmania, Tasmania, Australia

Supplemental tables

Table S1. The list of *Synechocystis* sp. PCC 6803 strains used in the study

Gene IDs are derived from the cyanobacterial genome database (cyanobase; http://genome.microbedb.jp/cyanobase).

**Strain**	**description**	**reference**
wild type (WT)	*Synechocystis* sp. PCC 6803 GT-P	[S1]
Δ*argD*	Ery^R replacement of *argD* (*slr1022*)	this study
f.argD$^+$/Δ*argD*	3xFLAG-tagged ArgD and Km^R replacement of *psbAII* in Δ*argD*	this study
f.astC$^+$/Δ*argD*	3xFLAG-tagged AstC and Km^R replacement of *psbAII* in Δ*argD*	this study
Δ*cphB*	Sp^R replacement of *cphB* (*slr2001*)	this study
f.argD$^+$/Δ*cphB*	3xFLAG-tagged ArgD and Km^R replacement of *psbAII* in Δ*cphB*	this study
f.gun4$^+$/Δ*gun4*	3xFLAG-tagged Gun4 (Sll0558) and Km^R replacement of *psbAII* in Δ*gun4* (Zeo^R)	[S2]

Table S2. The list of the specific binding partners of f.ArgD

The f.ArgD pull-down was separated by two dimensional blue native/SDS-PAGE (Figure 1B). The most intense protein spots were cut, trypsin-digested and analysed by protein mass-spectrometry.

protein name	gene	locus	protein MW (kDa)	coverage (%)
N-acetylornithine aminotransferase	*argD*	slr1022	46.567	57.6
Cyanophycinase	*cphB*	slr2001	29.389	72.3
Genome Uncoupled 4	*gun4*	sll0558	26.465	74.2
Slr0983	*rfbF*	slr0983	28.793	64.1

Table S2 supplements Figure 1B.

Table S3. **The list of the specific binding partners of f.Gun4**

The f.Gun4 pull-down was separated by SDS-PAGE. After staining the gel (Figure 2C), the most intense protein bands were cut, trypsin-digested and analysed by protein mass-spectrometry.

**protein name**	**gene**	**locus**	**protein MW (kDa)**	**coverage (%)**
pyruvate kinase 1	*pyk-1*	sll0587	51.96	44
N-acetylornithine aminotransferase	*argD*	slr1022	46.567	49.7
Genome Uncoupled 4	*gun4*	sll0558	26.465	60
Cyanophycinase	*cphB*	slr2001	29.389	72.3

Table S3 supplements Figure 2A.

Table S4. Metabolic changes caused by ornithine (Orn) or N-acetylornithine (AcOrn)-feeding

Synechocystis grown in BG-11 media were supplemented with 100 µM Orn or AcOrn for 40 min. The intracellular, mM concentrations of the metabolites were determined by LC-MS combined with GC-MS. Control-1, 2 and 3 correspond to the biologically independent samples measured before the addition of Orn or AcOrn. GABA, 2-aminobutyrate; 2-OG, 2-oxoglutarate; Ile, isoleucine; Asn, asparagine; Met, methionine; Glu, glutamate; Phe, phenylalanine; Gln, glutamine; Trp, tryptophan.

	control-1	control-2	control-3	Orn-1	Orn-2	Orn-3	AcOrn-1	AcOrn-2	AcOrn-3
fumarate	0.75	0.5	0.77	0.69	0.41	0.4	0.51	0.37	0.53
succinate	3.15	2.76	3.45	2.37	1.53	1.66	2.47	2.26	2.46
alanine	1.49	1.36	1.8	2.18	2.08	2.05	1.43	1.29	1.52
glycine	0.33	0.25	0.37	0.49	0.39	0.51	0.25	0.28	0.31
GABA	0.04	0.04	0.1	0.02	0.01	0.05	0.03	0.03	0.07
valine	0.24	0.35	0.72	0.2	0.15	0.21	0.11	0.1	0.19
2-OG	0.98	0.72	0.92	0.41	0.3	0.3	0.72	0.61	0.76
Ile	0.22	0.25	0.46	0.18	0.13	0.17	0.12	0.11	0.19
leucine	0.09	0.10	0.20	0.09	0.06	0.08	0.06	0.06	0.09
threonine	0.33	0.31	0.41	0.39	0.38	0.37	0.27	0.29	0.36
serine	1.34	1.43	1.48	1.23	1.19	1.20	1.25	1.27	1.31
proline	0.15	0.25	0.24	8.81	13.76	14.43	0.96	1.53	1.06
Asn	0.17	0.17	0.21	0.25	0.20	0.19	0.19	0.19	0.21
malate	2.98	2.41	3.46	3.05	2.16	2.08	2.50	2.01	2.57
citric	1.66	1.78	1.92	1.45	1.12	1.26	1.45	1.28	1.16
aspartate	0.44	0.58	0.53	0.97	0.82	0.69	0.40	0.41	0.34
Met	0.08	0.09	0.11	0.09	0.08	0.08	0.07	0.08	0.09
Glu	16.61	16.59	18.22	35.27	31.07	29.48	18.06	18.12	20.03
Phe	0.13	0.11	0.14	0.10	0.07	0.07	0.10	0.10	0.12
Gln	0.29	0.44	0.36	0.58	0.56	0.39	0.43	0.39	0.43
Orn	0.06	0.05	0.05	19.63	17.02	16.08	0.32	0.22	0.21
lysine	0.11	0.08	0.15	0.27	0.14	0.17	0.10	0.08	0.10
tyrosine	0.19	0.15	0.21	0.19	0.17	0.14	0.18	0.17	0.18
Trp	0.03	0.03	0.04	0.03	0.02	0.02	0.03	0.04	0.04
arginine	0.07	0.08	0.08	0.71	0.67	0.69	0.09	0.10	0.10
citrulline	0.16	0.13	0.10	5.88	5.63	5.07	0.49	0.52	0.45
AcOrn	n.d.	n.d.	n.d.	0.19	0.15	0.15	n.d.	n.d.	n.d.

Table S4 contains the complete data used for generating the heat map shown in Figure S3A.

Table S5. Oligonucleotides used in the study.

primer	sequence	reference
Primer for the replacement of *argD* with erythromycin resistance cassettes (forward-1):	GGCTTCGGTGAAACGATTTAC	This study
Primer for the replacement of *argD* with erythromycin resistance cassettes (forward-2):	GCATCCCTTAACTTGTTTTTCGGCG ATCGCCACTTTGGTTTG	This study
Primer for the replacement of *argD* with erythromycin resistance cassettes (reverse-1):	CTGCAATCTGATGCGATTATTGAAC AACAGGGGAATAGGTCACG	This study
Primer for the replacement of *argD* with erythromycin resistance cassettes (reverse-2):	GCCGAGCCGGAATGAGCAGA	This study
Primer for the replacement of *cphB* with spectinomycin resistance cassettes (forward-1):	CAACCCCAGCGTCCCGTAAC	This study
Primer for the replacement of *cphB* with spectinomycin resistance cassettes (forward-2):	GAAGTAATCGCAACATCCGCGGAT ATGGCGGTGGGGCTG	This study
Primer for the replacement of *cphB* with spectinomycin resistance cassettes (reverse-1):	GAAGTCGAGGCATTTCTGTCCGGT TGGGAGGATAGGGGCAT	This study
Primer for the replacement of *cphB* with spectinomycin resistance cassettes (reverse-2):	GCGCAACACGCCGACTATC	This study
Primer for cloning *f.argD*$^{+}$/Δ*argD (forward):*	AGCTACATATGACCTATTCCCCTGT TGTT	This study
Primer for cloning *f.argD*$^{+}$/Δ*argD (reverse):*	GGATCAGATCTCAAACCAAAGTGG CGAT	This study
Primer for cloning *f.astC*$^{+}$/Δ*argD (forward):*	CTAGACGCGGCCGCAGCAATTGAA CAAACAGCAATTACA	This study
Primer for cloning *f.astC*$^{+}$/Δ*argD (reverse):*	GAGGACAGATCTTTACGCCCCAAC CACCTTCG	This study
Primer for amplifying FLAG-constructs (forward):	TGTCATCTATAAGCTTCGTG	This study
Primer for amplifying FLAG-constructs (reverse):	ATCCGCCGGCAGACGTTCTTCC	This study
Primer for sequencing FLAG-constructs (forward):	AACTCTCATTAATCCTTTAG	This study
Primer for sequencing FLAG-constructs (reverse):	AGACCGATACCAGGATCTTG	This study

Supplemental figures

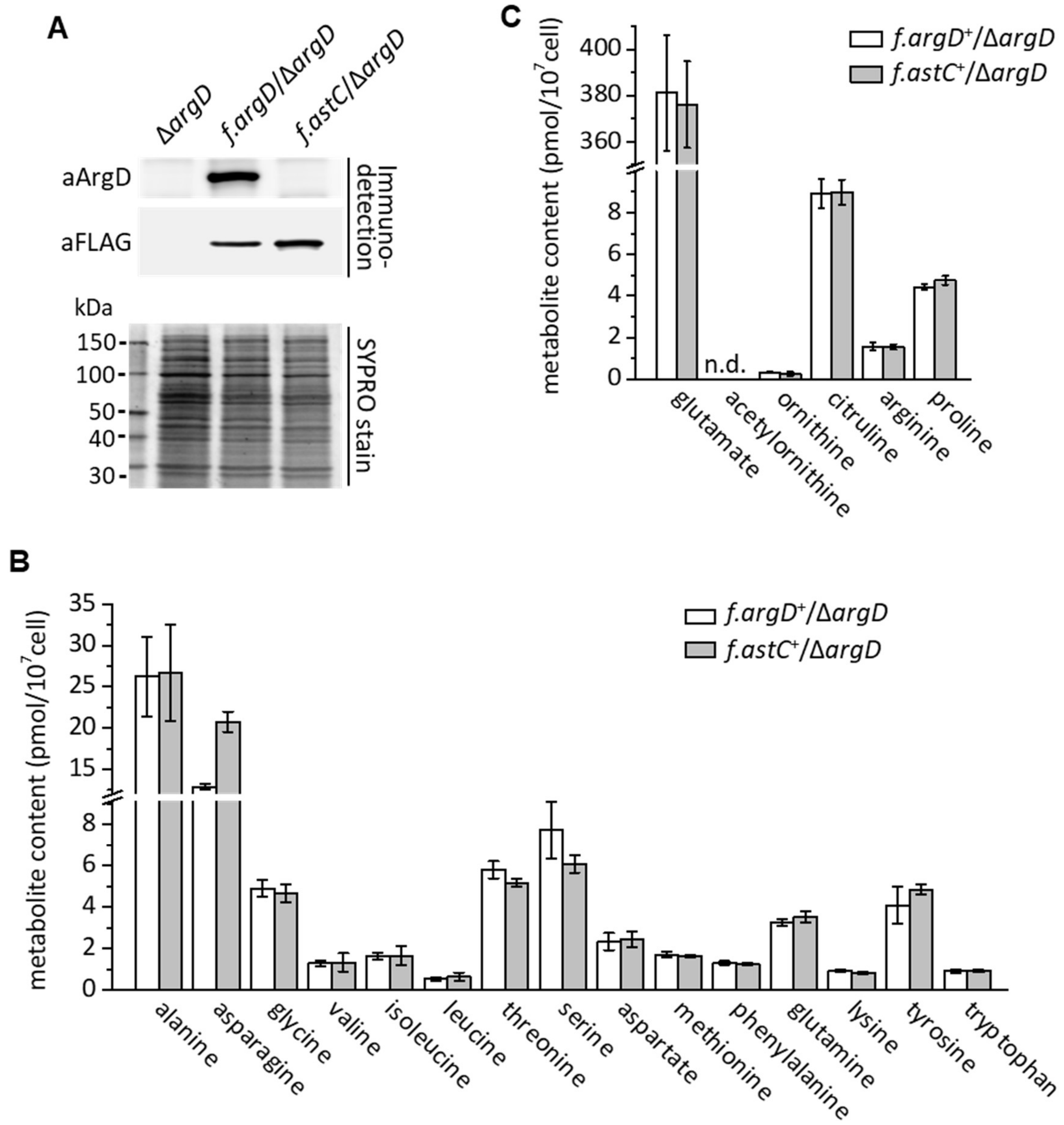

Figure S1. Functional replacement of *Synechocystis* ArgD with *E. coli* AstC

(A) Levels of FLAG-tagged ArgD (f.ArgD) and FLAG-tagged AstC (f.AstC) in the cytosol isolated form the *f.argD*$^+$/Δ*argD* and *f.astC*$^+$/Δ*argD* strains. The f.ArgD and f.AstC proteins were detected using antibody against their 3xFLAG-tag sequence. The SYPRO Orange dye of the SDS gel is shown for loading control.

(B) The cellular content of selected metabolites in *f.argD*$^+$/Δ*argD* (white) and *f.astC*$^+$/Δ*argD* (grey) was determined by LC-MS and GC-MS analysis. The columns and error bars represent the averaged data and standard deviation derived from samples collected from three independent, exponentially grown cultures, respectively.

(C) Cellular content of the Arg metabolism-related amino acids in *f.argD*$^{+}$/Δ*argD* (white) and *f.astC*$^{+}$/Δ*argD* (grey). For details see panel (B). n.d., not detected.

Figure S1 supports Figures 1A, 2C and D by indicating that the f.AstC is expressed in Δ*argD* and it fulfills the enzymatic function of ArgD in the biosynthesis of Orn without significantly modifying the accumulation of amino acids.

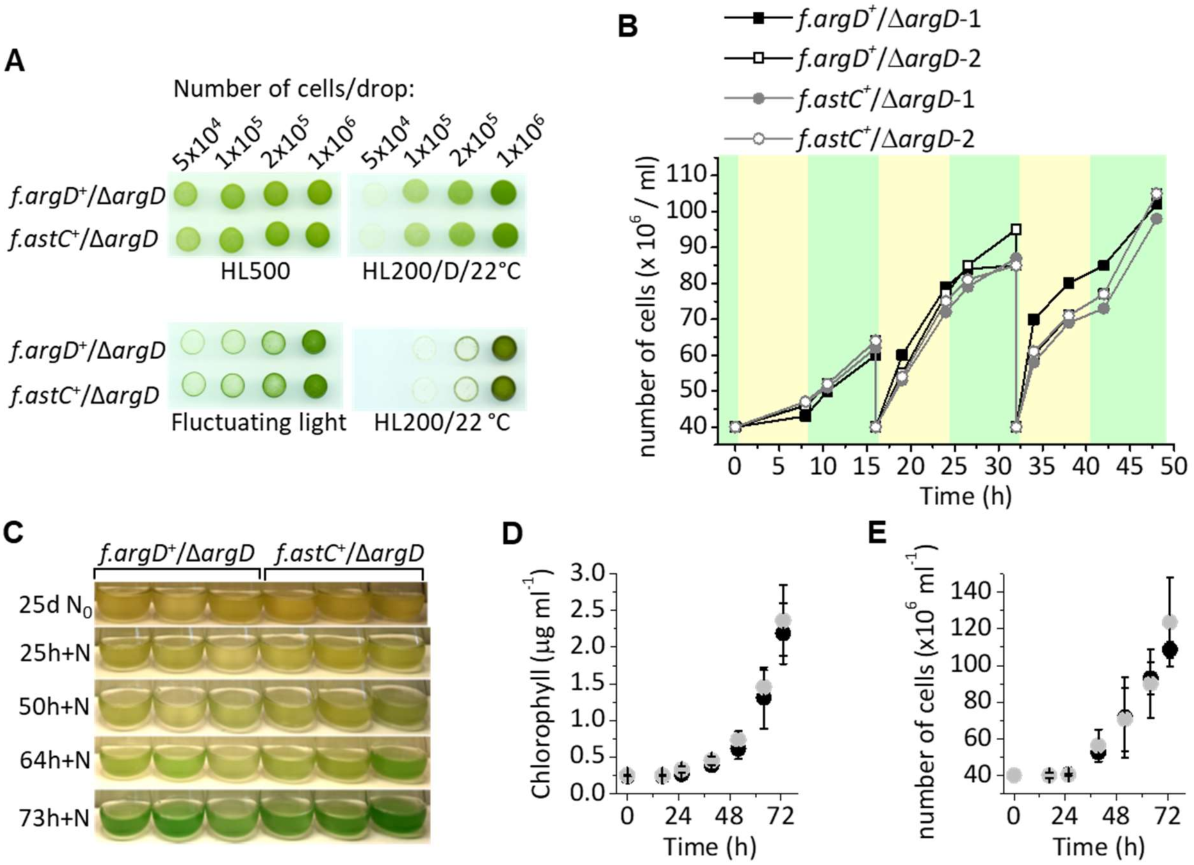

Figure S2. Comparison of the *f.argD*$^+$/Δ*argD* and *f.astC*$^+$/Δ*argD* strains under various physiological conditions

(A) Growth of *f.argD*$^+$/Δ*argD* and *f.astC*$^+$/Δ*argD* monitored in plate-drop experiments using the following conditions: HL500, continuous illumination with 500 µmol m^{-2} s^{-1}; fluctuating light, 5 min dark / 5 min 500 µmol m^{-2} s^{-1}; HL200/D/22 °C and HL200/22 °C, 200 µmol m^{-2} s^{-1} light intensity combined with 22 °C cold stress during diurnal growth (12/12 h) or continuous light, respectively.

(B) Proliferation of *f.argD*$^+$/Δ*argD* and *f.astC*$^+$/Δ*argD* cells in liquid medium during repeated changes of N-rich (green background) and N-less (yellow background) media. When the medium was changed to the N-less, the amount of cells in every culture was adjusted to 40x10^6 cells / ml. The duplicate cultures are designated by 1 and 2.

(C) Resuscitation of *f.argD*$^+$/Δ*argD* and *f.astC*$^+$/Δ*argD* cultures. Dormancy was induced by 25 d of N-deprivation. After 25 d in N_0 media the cells were washed to BG-11 (+N) to reach 40 x 10^6 cells/ml concentration. Cultures were monitored at the indicated time points.

(D) and (E) are the averaged Chl and cell contents of the *f.argD*$^+$/Δ*argD* (black symbols) and *f.astC*$^+$/Δ*argD* (grey symbols) cultures described in panel (C), respectively.

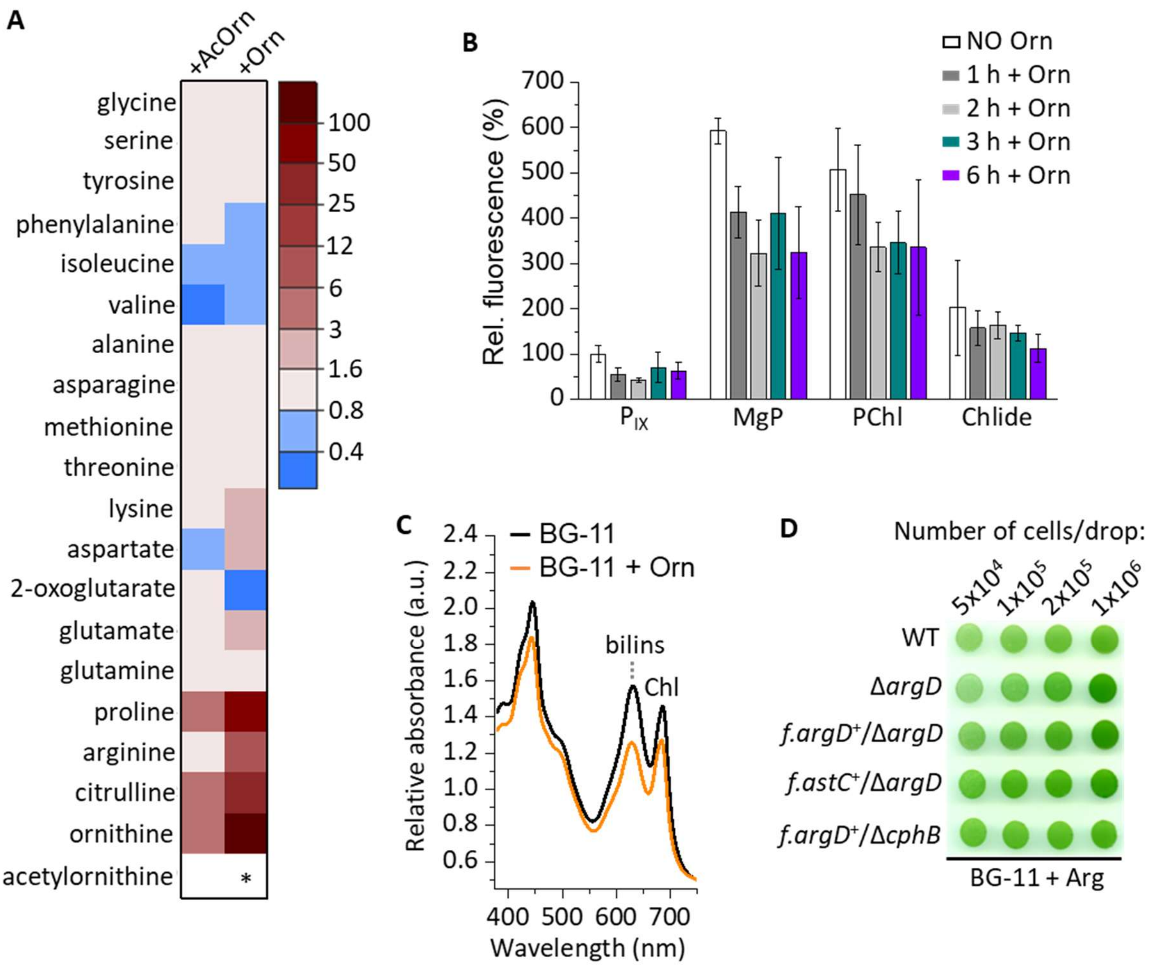

Figure S3. Response to Arg, N-acetylornithine (AcOrn) or ornithine (Orn) feeding

(A) *Synechocystis* cultures were supplemented with 100 µM AcOrn or Orn for 40 min. The cellular content of the selected metabolites was determined by LC-MS and GC-MS analysis, and is shown after normalization to the control sample (without the added ornithines). The heat map was generated from the averaged data obtained from three independent biological experiments. *, AcOrn was not detected in the control sample, even after AcOrn-feeding; only after the addition of Orn (163 ± 26 µM). The complete dataset is shown in the Table S4.

(B) Changes in the relative abundances of the biosynthetic intermediates of Chl in Orn-fed WT cells. Samples were taken before (NO Orn) and after the addition of 1 mM Orn at the indicated time points.

(C) The effect of Orn on the pigmentation of *Synechocystis* WT cell. The cell absorption spectra were measured on same amount of cells cultivated for 3 d in BG-11 with (orange line) or without (black line) 1 mM Orn.

(D) The effect of Arg on the growth of the *Synechocystis* strains used in the study.

Figure S3 complements Figures 3, and explains the choice of Orn feeding for the purpose of perturbation of Arg metabolism and its effect on the *in vivo* accumulation of the Gun4-ArgD-CphB complex.

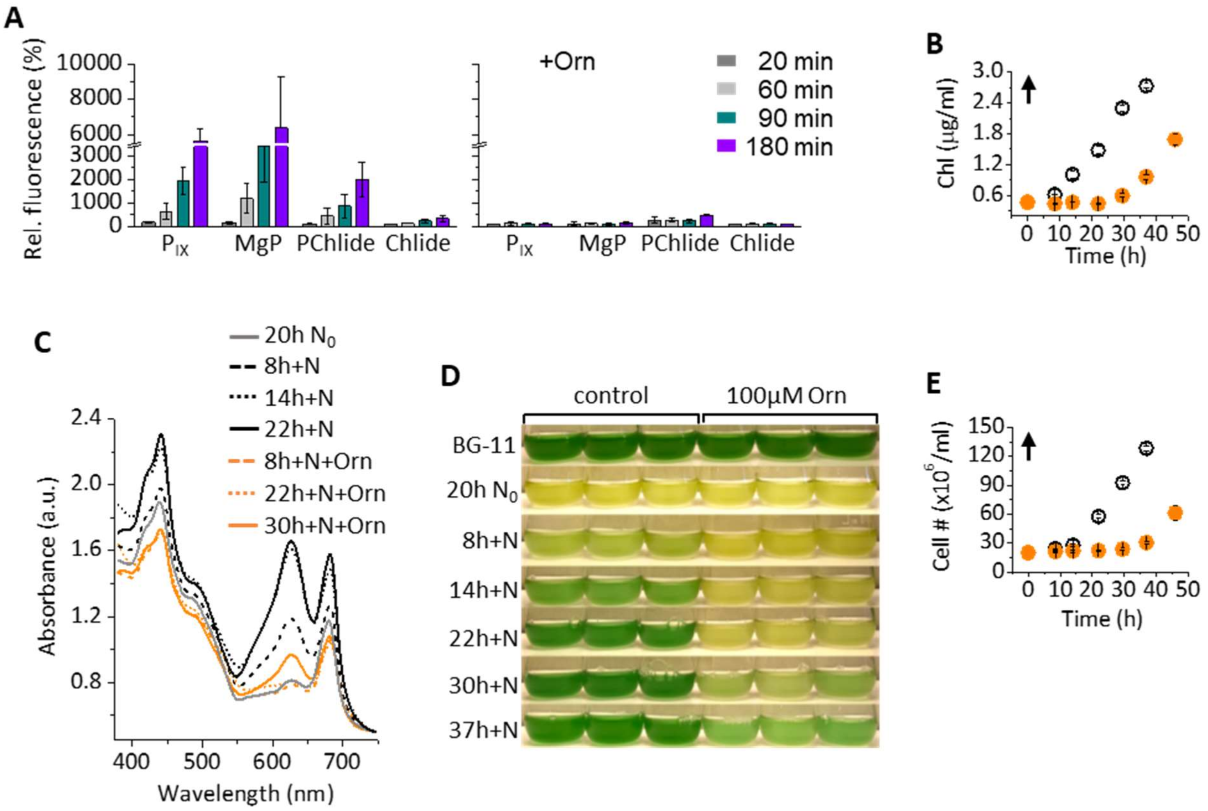

Figure S4. Orn postpones the recovery of *Synechocystis f.argD*$^+$/Δ*argD* cells from nitrogen-deprivation

(A) The relative amounts of Chl precursors were measured during N-repletion of 20 h N-starved *f.argD*$^+$/Δ*argD* cells. The amounts before the addition of 1mM $NaNO_3$ or 1mM $NaNO_3$ + 100 µM Orn (+Orn) were taken as 100 %. Columns and error bars represent the averaged data and standard deviation of three independent experiments, respectively.

(B) Accumulation of Chl in *f.argD*$^+$/Δ*argD* cultures during N-repletion in the absence (black) or presence (orange) of 100 µM Orn. Error bars represent standard deviation of three independent experiments. The time of N-upshift is marked by upward arrows.

(C) Accumulation of phycobilin (625nm) and Chl-binding (682 nm) proteins in the 20 h N-starved *f.argD*$^+$/Δ*argD* cells before (20h N_0) and after the addition of 1 mM $NaNO_3$ without (black) or with (orange) 100 µM Orn. The *in vivo* absorption spectra were measured on the same amount of cells at indicated time points.

(D) The cultures before (BG-11) and after (20 h N_0) N-starvation, and during the re-greening without (control) or with 100 µM Orn (as described in panel A) were photographed at indicated times.

(E) Changes in the concentration of cells in the cultures described in panel A and B.

Figure S4 supplements Figure 4, and shows that similarly to the WT control, the *f.argD*$^+$/Δ*argD* cultures exhibit delayed re-greening in the presence of Orn.

Supplemental references

S1. Tichý, M., Bečková, M., Kopečná, J., Noda, J., Sobotka, R., and Komenda, J. (2016). Strain of *Synechocystis* PCC 6803 with aberrant assembly of photosystem II contains tandem duplication of a large chromosomal region. Front Plant Sci *7*, 648. 10.3389/fpls.2016.00648.

S2. Sobotka, R., Dühring, U., Komenda, J., Peter, E., Gardian, Z., Tichý, M., Grimm, B., and Wilde, A. (2008). Importance of the cyanobacterial GUN4 protein for chlorophyll metabolism and assembly of photosynthetic complexes. J Biol Chem *283*, 25794-25802. 10.1074/jbc.M803787200.
